# Kinetic analysis of the influenza A virus HA/NA balance reveals contribution of NA to virus-receptor binding and NA-dependent rolling on receptor-containing surfaces

**DOI:** 10.1371/journal.ppat.1007233

**Published:** 2018-08-13

**Authors:** Hongbo Guo, Huib Rabouw, Anne Slomp, Meiling Dai, Floor van der Vegt, Jan W. M. van Lent, Ryan McBride, James C. Paulson, Raoul J. de Groot, Frank J. M. van Kuppeveld, Erik de Vries, Cornelis A. M. de Haan

**Affiliations:** 1 Virology Division, Department of Infectious Diseases and Immunology, Faculty of Veterinary Medicine, Utrecht University, Utrecht, the Netherlands; 2 Laboratory of Virology, Wageningen University and Research, Droevendaalsesteeg 1, PB Wageningen, the Netherlands; 3 Departments of Cell and Molecular Biology, Chemical Physiology, and Immunology and Microbial Science, Scripps Research Institute, La Jolla, California, United States of America; Emory University School of Medicine, UNITED STATES

## Abstract

Interactions of influenza A virus (IAV) with sialic acid (SIA) receptors determine viral fitness and host tropism. Binding to mucus decoy receptors and receptors on epithelial host cells is determined by a receptor-binding hemagglutinin (HA), a receptor-destroying neuraminidase (NA) and a complex *in vivo* receptor-repertoire. The crucial but poorly understood dynamics of these multivalent virus-receptor interactions cannot be properly analyzed using equilibrium binding models and endpoint binding assays. In this study, the use of biolayer interferometric analysis revealed the virtually irreversible nature of IAV binding to surfaces coated with synthetic sialosides or engineered sialoglycoproteins in the absence of NA activity. In addition to HA, NA was shown to be able to contribute to the initial binding rate while catalytically active. Virus-receptor binding in turn contributed to receptor cleavage by NA. Multiple low-affinity HA-SIA interactions resulted in overall extremely high avidity but also permitted a dynamic binding mode, in which NA activity was driving rolling of virus particles over the receptor-surface. Virus dissociation only took place after receptor density of the complete receptor-surface was sufficiently decreased due to NA activity of rolling IAV particles. The results indicate that *in vivo* IAV particles, after landing on the mucus layer, reside continuously in a receptor-bound state while rolling through the mucus layer and over epithelial cell surfaces driven by the HA-NA-receptor balance. Quantitative BLI analysis enabled functional examination of this balance which governs this dynamic and motile interaction that is expected to be crucial for penetration of the mucus layer and subsequent infection of cells by IAV but likely also by other enveloped viruses carrying a receptor-destroying enzyme in addition to a receptor-binding protein.

## Author summary

Influenza A virus (IAV) tropism is largely determined by the interaction of virus particles with the sialic acid receptor repertoire of the host. IAVs encounter a diverse range of sialic acid receptors that can function as decoys (e.g. in the mucus that covers epithelial cells) or as entry receptors. We studied the dynamics of IAV-receptor interactions in real-time using biolayer interferometry (BLI) in combination with synthetic glycans and recombinant sialoglycoproteins mimicking *in vivo* receptors. Thereby we could show that IAVs do not continuously associate and dissociate with receptor-coated surfaces but actually were rolling over the surface with which they remained permanently associated until the receptors were sufficiently cleared. This required the concerted action of the receptor-binding hemagglutinin (HA) and the receptor-destroying neuraminidase (NA) on the receptor surface. We could quantify the precise HA-NA-receptor balance that determined the speed of rolling and eventual elution from the surface by BLI and propose a model in which IAV is permanently, but dynamically, associated with receptors on mucus or host cells *in vivo*.

## Introduction

Specificity, avidity and dynamics of influenza A virus (IAV)-receptor interactions are determining factors in host tropism and pathogenesis. Virus attachment to sialic acid (SIA) receptors on host cell surfaces and decoy mucins is mediated by hemagglutinin (HA) [1–3], while neuraminidase (NA) removes receptors by cleaving SIAs [4–6]. A precisely tuned functional HA/NA balance [7–15] is required for efficient infection of, and replication in, a specific host.

HA and NA properties affect host- and cell-specificity but have been studied in much more detail for HA because of the relative lack of accurate NA assays [16]. IAVs that infect humans bind preferentially to α2,6 sialosides, having an α2,6-linkage between SIA moieties and the penultimate residue, whereas avian IAVs prefer binding to α2,3-linked SIAs [17–19]. Cell surface glycan composition, as well as branching, modification and linkage-type of the internal carbohydrate chain residues, is variable between host and cell type and also strongly affects binding affinity (Reviewed in [20]). NAs of avian IAVs are highly active and prefer cleavage of α2,3-linked sialoglycans, while human virus NAs cleave α2,3- and α2,6-linked SIAs with lower activity [21–23].

The HA/NA balance is important for initiating IAV infection. Abundantly sialylated mucins in the mucus layer covering the airway epithelial cells bind HA and may trap IAVs before they reach the epithelial cells [24,25]. This needs to be counteracted by NA activity sufficiently matching HA binding specificity. This is also required to prevent the virus-trapping at sites on the cell surface that do not support efficient endocytosis [26,27]. NA activity-dependent de-sialylation of the surface of the infected cell and the virus envelope glycoproteins facilitates budding and release of new virus particles and prevents virus aggregation [28]. As a consequence, viral fitness depends on continuous fine-tuning of the HA/NA balance during virus evolution. Replication of viruses, harboring mismatched pairs of HA and NA or propagated in the presence of inhibitory decoy receptors or NA inhibitors, could be rescued by adaptive mutations in the HA, NA or both proteins [7,11,13,29]. Cross-species transmission events, leading to human pandemics in the past, required adaptions in both HA and NA [10,30]. When infecting a novel host species, a non-optimal HA/NA balance is for instance adapted by evolving increased NA activity as well as decreased binding by HA to the decoy glycan-receptor repertoire on mucins present in the mucus, and which differs between different hosts. [29,31–33].

There is no straightforward method to determine the HA/NA balance of IAVs. Hemagglutination of erythrocytes, which harbor poorly defined receptor repertoires, does not well reflect binding avidity and dynamics. Current setups of solid phase binding assays (e.g. ELISAs, glycan arrays) as endpoint binding assays do not handle well the polyvalent aspects of virus binding by masking the dynamics of virus binding, even more so as precise variation of receptor density is difficult. Moreover, besides a wide range of synthetic glycans, only very few glycoproteins (mostly fetuin) are being employed in current binding studies, thus limiting studies on the effects of diversity and clustering of glycans as naturally presented on glycoproteins. NA activity assays often use soluble substrates poorly reflecting natural sialosides [5,34]. The soluble substrate 2′-(4-methylumbelliferyl)-α-d-N-acetylneuraminic acid (MUNANA) can be used to determine the NA activity on a soluble substrate but ignores the effects of the virion context on NA activity on a receptor-coated surface (e.g. binding of the virion to the polyvalent substrate via its HA). Thus, methods that determine the dynamic effects of HA and NA activity urgently need improvement to allow integration into a model that accurately provides a quantitative description of the dynamic interaction between IAV particles and (decoy) receptors.

Biolayer interferometry (BLI) is increasingly being used for analyzing virus-receptor interactions [35–38], but standard methods for quantifying kinetic parameters describing IAV-receptor interactions are lacking. Here we have used this label-free real-time binding analysis method to study the dynamics of IAV-receptor interactions. Our results indicate that the initial virus binding rate is the prime, and physiologically most important kinetic parameter of HA-dependent IAV particle binding to synthetic as well as natural glycoprotein receptors. NA was shown to contribute to virus binding and to be absolutely required for virus dissociation. In turn, NA activity is critically dependent on the ability of virions to bind to a receptor-coated surface. The HA/NA balance of different virus-receptor combinations was determined by measuring empirical virus binding and elution parameters. Prior to virus elution, NA-dependent morphological changes in receptor-associated virus particles were observed as well as rolling of virus particles over a receptor-coated surface in a NA activity-dependent manner.

## Results

### IAV binding can be quantified by determination of the initial binding rate

BLI is a potentially versatile tool to obtain mechanistic and quantitative insight into the dynamics of virus-receptor interaction by real-time recording of virion binding to, and release from, receptor-coated surfaces. These interactions are highly polyvalent by nature and expected to be poorly described by equilibrium binding models. In addition, receptor density and identity are key determinants of the interaction. We therefore established a flexible experimental set-up, using synthetic sialoside receptors as well as N-linked or O-linked sialoglycoproteins which carry sialoglycan receptors attached in the natural linkage-conformation that is encountered *in vivo*. We first evaluated the applicability of the set-up to a quantitative description of IAV-receptor binding kinetics. To assist interpretation of the results, the approximate geometric properties of a streptavidin (SA) BLI sensor surface, to which biotinylated glycans or glycoproteins can be tightly attached (*K*_d_ = 10^−14^), and of IAV virions are depicted in S1 Fig. Under the assumption that 10% of the virion surface can interact with the sensor surface (S1B Fig), maximally seven HA trimers can interact simultaneously with receptor-loaded streptavidin (S1C Fig).

**Fig 1:**
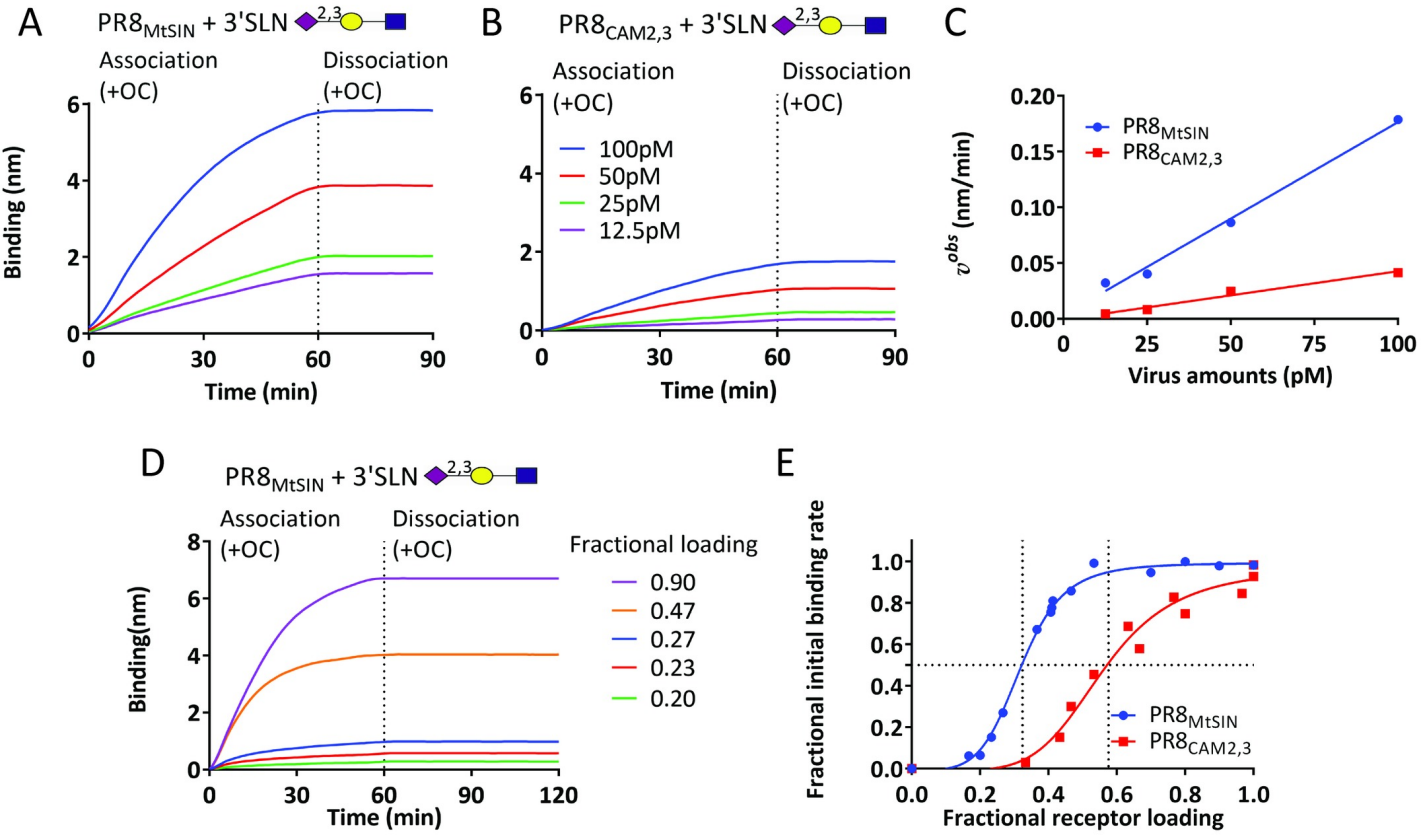
Determination of IAV binding rates by BLI. SA sensors were fully loaded with biotinylated Neu5Acα2-3Galβ1-4GlcNAcβ-Sp8 (3’SLN). PR8_MtSIN_ (A) and PR8_CAM2,3_ (B) were bound (12.5 pM to 100 pM, corresponding to 0.75E+09 to 6E+09 particles in 100 μl; S7 Fig) in the presence of 10 μM OC. After 60 minutes sensors were washed (PBS, 3 times) and virus dissociation in PBS was recorded for 30 minutes. (C) *v*^*obs*^ of both viruses was plotted against virus concentration. The resulting linear curves (P<0.001 as determined by IBM SPSS statistic 24) as well as *v*^*obs*^ normalized for particle number (mean ± SD]; 0.19285 ± 0.044309 for PR8_MtSIN_ and 0.03985 ± 0.007378 nm/min per 100pM virus particles for PR8_CAM2,3_; P = 0.0005) differed significantly. (D) 3’SLN receptor was loaded to a range of densities and allowed to bind to 100 pM virus (PR8_MtSIN_) in the presence of OC. After 60 minutes sensors were washed (PBS, 3 times) and virus dissociation in PBS was recorded for 60 minutes. (E) The *v*^*obs*^ was calculated for all binding curves of PR8_MtSIN_ and PR8_CAM2,3_ (shown in S4 Fig) and plotted as fractional binding rate (y-axis) against fractional receptor loading density (x-axis).

First, binding of two IAV lab strains carrying a different HA (PR8_MtSIN_ and PR8_CAM2,3_; S2 Fig and S3 Fig provide an overview of the viruses and receptors used in this paper) to the short synthetic glycan 3’SLN (Neu5Acα2-3Galβ1-4GlcNAcβ) was analyzed (Fig 1). Virus association was performed in the presence of the NA inhibitor oseltamivir carboxylate (OC) and the lack of virus dissociation after transfer of sensors to buffer containing OC (Fig 1A and Fig 1B) showed that virus binding is virtually irreversible (off-rate constant *k*_off_ ≈ 0) due to highly polyvalent interactions between virus particles and the receptor-coated BLI sensor surface. PR8_MtSIN_ is a faster binder than PR8_CAM2,3_ (Fig 1A and Fig 1B), but reached a maximum binding signal of ~9 nm at the highest concentration only after 4 hours (S4A Fig and S4B Fig). A fully occupied sensor surface theoretically accommodates 3.3E+07 spherical virus particles (100 nm diameter; S3 Fig and [39–42]). This fitted well with the experimentally determined number of virus particles bound to a maximally loaded sensor as determined by quantification of NA activity (S4C Fig). Importantly, sensor-regeneration followed by re-binding of virus from the same well could be consecutively repeated at least 6 times yielding identical curves (S5 Fig). Thus, substantial virus decay during the assay or binding of a limited subset of virus particles with particular properties did not occur.

Analysis of IAV-receptor interactions has predominantly been focused on quantification of an apparent dissociation constant (*K*_*D*_ = *k*_off_/*k*_on_) by measuring polyvalent binding of virus particles or artificial HA polymers (e.g. by antibody complexation) after a fixed binding time to geometrically poorly characterized receptor-coated surfaces with often unknown receptor density and orientation. However, as virus binding is virtually irreversible, equilibrium binding models do not apply and the binding rate equation (*v* = *k*_on_[virus][R]—*k*_off_[R.virus]) can be simplified to *v* = *k*_on_[virus][R]. [Virus] is nearly constant (as shown by repeated binding experiments) and receptor concentration [R] on a receptor-coated surface can be regarded as a constant during the initial binding phase. Indeed, the expected direct proportionality between the observed initial observed binding rate (*v*^*obs*^ = dB/dt = *k*_on_[virus][R]) and virus concentration was observed for the fast (PR8_MtSIN_; r = 0.997, P = 0.0002) and the slow binding (PR8_CAM2,3_; r = 0.9915, P = 0.0009) virus (Fig 1C).

The effect of receptor density on IAV association and dissociation rates can be precisely determined as BLI provides quantitative recording of receptor loading levels. Lowering the receptor density, resulting in less potential HA-receptor contacts between virus and sensor surface, gradually reduced maximum binding levels (Fig 1D and S4D and S4E Fig) as well as the *v*^*obs*^ (Fig 1E). Remarkably, irreversible binding was observed at any receptor density that supported binding (Fig 1D). A ~2-fold higher fractional receptor loading was required for the weaker binder PR8_CAM2,3_ (0.593±0.01752) than for PR8_MtSIN_ (0.332±0.00364) to reach 50% of the maximum fractional initial binding rate (P<0.001) (Fig 1E). PR8_MtSIN_ reached a maximum binding rate at ~55% receptor loading whereas the weaker binder PR8_CAM2-3_ reached its maximum binding rate close to maximum receptor density. A ~3-fold reduction in receptor loading was already sufficient to reduce the *v*^*obs*^ from maximal to zero for both viruses. This suggests a narrow margin between the number of interacting HA-receptor pairs, below which binding is undetectable due to high reversibility (high *K*_D_) and above which binding is irreversible (see Fig 1D, no dissociation at any receptor density). We conclude that *v*^*obs*^, which is directly related to virus concentration and receptor density, is the most suitable parameter for quantification of virus binding strength over a range of receptor densities and is likely to reflect *in vivo* virus binding, which probably occurs to levels very far from saturation.

### Virus concentration-independent determination of relative receptor-specificity

**Fig 2:**
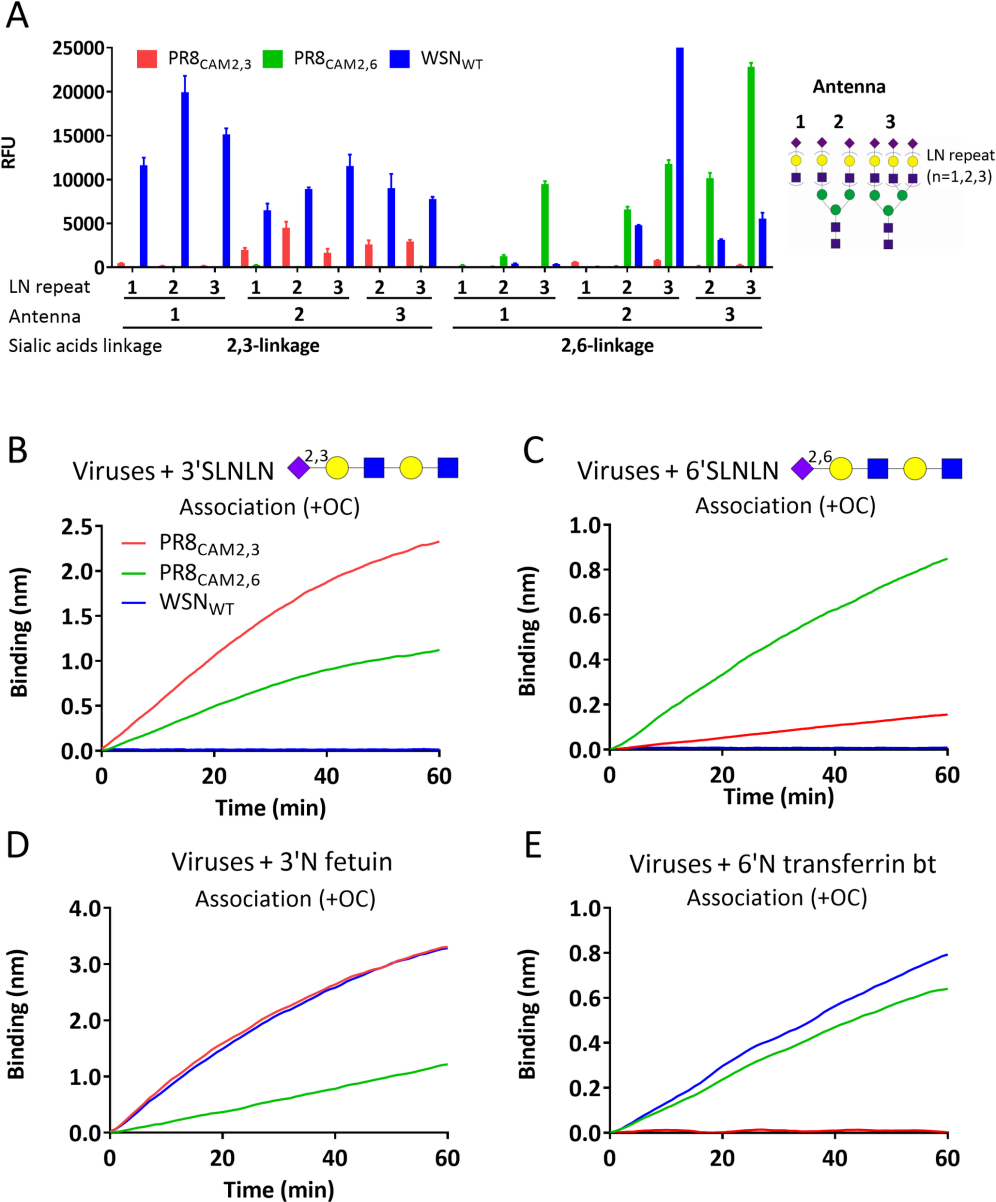
Quantification of virus binding specificity by BLI. (A) Specificity of recombinant soluble HA trimers of PR8_CAM2,3_, PR8_CAM2,6_ or WSN_WT_ was determined by glycan array analysis. Binding to mono-, bi- and tri-antennary glycans carrying one, two or three LacNAc repeats terminated with a α2,3- or α2,6-linked SIA is indicated. Means of 6 independent replicates are graphed, standard errors of the means are indicated. Biotinylated 3’SLNLN or 6’SLNLN (B, C), Fc-tagged 3’N fetuin (D) or biotinylated 6’N transferrin (6’N transferrin bt) (E) were loaded to maximum levels to SA or Protein A biosensors after which binding of 100 pM PR8_CAM2,3_, PR8_CAM2,6_ or WSN_WT_ virus was monitored for 60 minutes in the presence of 10 μM OC.

As the initial binding rate is linear with virus concentration, the relative specificity for two receptors (*v*^*rel*^ = *v*^*obs1*^/ *v*^*obs2*^) is independent of virus concentration and the relative-binding specificity of viruses can therefore be quantitatively compared without the need for elaborate virus quantitation procedures. As an example, we quantified the effect on receptor-specificity of amino acid substitution E190D in HA of PR8 which is predicted to cause a shift in binding from α2,3- to α2,6-linked SIA receptors in H1 [43]. Indeed glycan array analysis of the HA proteins of lab strains PR8_CAM2,3_ and PR8_CAM2,6_, which only differ by substitution D190E, showed an absolute specificity shift (Fig 2A). In contrast, virus binding quantified by BLI showed a relative specificity shift (*v*^*rel*^ = *v*^*3’SLNLN*^/*v*^*6’SLNLN*^) of 19.7±0.11 for PR8_CAM2,3_ to 1.2±0.18 for PR8_CAM2,6_ (Fig 2B and Fig 2C). Remarkably, strain WSN HA, which carries 190E plus some additional mutations in comparison to PR8_CAM2,3_, displayed efficient binding to α2,3- as well as α2,6-linked synthetic glycans on the microarray, but WSN_WT_ virus did not bind to synthetic glycans in the BLI assay. Possibly, soluble HA proteins can have access to short glycans on glycan arrays whereas virus envelope-embedded HAs cannot, depending on virus strain, bind to the same glycans on BLI sensors.

To test this, we examined binding to glycoproteins carrying α2,3- or α2,6-linked SIAs on N-linked sialoglycans (3’N fetuin or 6’N transferrin bt; Fig 2D and Fig 2E). PR8_CAM2,3_ virus displayed absolute specificity for 3’N fetuin. However, an absolute α2,3- to α2,6-linked SIA specificity shift by mutation E190D, as observed above by glycan array analysis, did not occur for virus binding to glycoproteins (*v*^rel^ = *v*^3’N fetuin^/*v*^6’N transferrin^ = 1.4±0.21 for PR8_CAM2,6_), similar to what was observed for the synthetic glycans (Fig 2B and Fig 2C). Glycoproteins, in contrast to synthetic glycans, supported WSN_WT_ virus binding to BLI sensors which displayed dual binding specificity (*v*^rel^ = 4.9±0.15) in agreement with the glycan array results for WSN HA proteins. In conclusion, determination of *v*^*obs*^ allows quantification of relative receptor specificity differences for virus particles, which is independent of virus concentration (S4G Fig, S4H Fig and S4I Fig).

The application of (tailor-made) glycoproteins enables to elucidate quantitative differences in IAV receptor-binding fine specificity, which is a step forward to mimicking virus-receptor interactions that occur *in vivo*. The versatility of the latter is further illustrated by showing the relative specificity for N-linked versus O-linked glycans, using engineered fetuin constructs (S2 Fig). O-linked glycans are abundantly present on soluble mucins present in respiratory mucus. As an initial experiment to explore potential differences in binding dynamics to O- or N-linked glycans we expressed fetuin variants containing three N-linked and/or three O-linked glycans and compared the binding of PR8_CAM2,3_ and PR8_MtSIN_ to these receptors (S6A–S6D Fig). The glycan side chains present on these recombinant fetuins were confirmed by lectin binding assays with and without prior PNGase F treatment to remove N-glycans (S2D Fig and S6E–S6G Fig). Only PR8_CAM2,3_ appeared to be able to bind to 3’O Fetuin (S6C Fig). In agreement herewith, whereas PR8_MtSIN_ bound at a two-fold higher rate to 3’N fetuin than PR8_CAM2,3_ (S6B Fig), the presence of O-linked glycans in 3’N+O fetuin reduced the difference in binding rate (S6A Fig).

### NA activity on a receptor-coated surface requires virion binding

**Fig 3:**
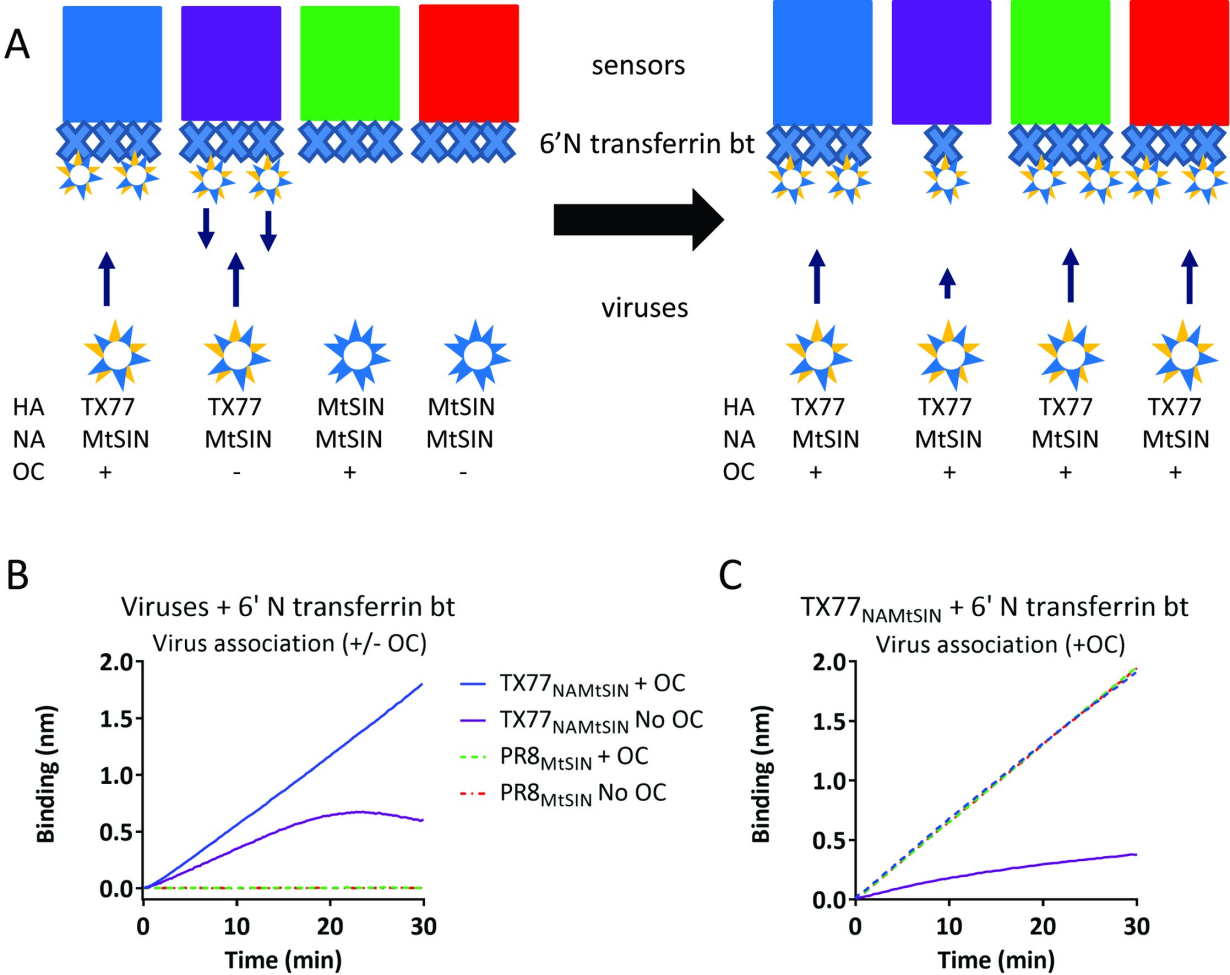
Receptor cleavage by virus particle-associated NA requires HA-dependent virus binding. (A) Schematic representation of the experimental set-up. Colours of the sensors correspond with the colours of the lines in (B) and (C). 6’N transferrin bt is indicated by the large Xs. Origin of the HA and NA proteins of the viruses used is indicated as well as the absence of presence of OC during the incubation of the sensors with the viruses. The left and right panels correspond with the graphs shown in (B) and (C), respectively. (B) Biotinylated 6’N transferrin bt was loaded to maximum levels on Streptavidin sensors and bound with 100 pM PR8_MtSIN_ or TX77_NAMtSIN_ in absence or presence of 10 μM OC as indicated. (C) Sensors bound in (B) with virus were regenerated at pH2, removing all virus particles, and subsequently bound again with 100 pM TX77_NAMtSIN_ to assess the extent of desialytion that occurred in (B) by neuraminidase activity.

NA activity contributes to the dynamic interaction of virions with receptor-coated surfaces. We first determined whether non-bound virions are able to cleave receptors loaded on BLI sensors. We used viruses carrying the same NA but a different HA (PR8_MtSIN_ and TX77_NAMtSIN_; S2 Fig, see Fig 3A for experimental set up) that bind α2,6-linked SIAs (TX77_NAMtSIN_) or α2,3-linked SIAs (PR8_MtSIN_) as demonstrated by the exclusive binding of TX77 _NAMtSIN_ to 6’N transferrin bt(Fig 3B). In the presence of NA activity, the binding rate of TX77 _NAMtSIN_ became increasingly lower in time, presumably reflecting virion release due to ongoing receptor depletion. Regeneration of sensors, which removes all virus particles but leaves the biotinylated glycans on the sensors, was followed by a second round of TX77_NAMtSIN_ binding in order to determine the extent of de-sialylation that occurred in the first round of binding (Fig 3C). Only TX77_NAMtSIN_ binding in the first round in absence of OC led to receptor desialylation as detected by inefficient binding of TX77_NAMtSIN_ in the second round. In contrast, α2,3 specific PR8_MtSIN_ did not bind to the sensor and thereby could not remove SIAs, as shown by efficient re-binding of TX77_NAMtSIN_ upon regeneration. We conclude that non-bound virions do not contribute to sialidase activity and do not have to be taken into account when studying the effect of virion-associated NA activity on IAV receptor binding dynamics by BLI.

### NA activity determines the dynamics of IAV-receptor interactions

**Fig 4:**
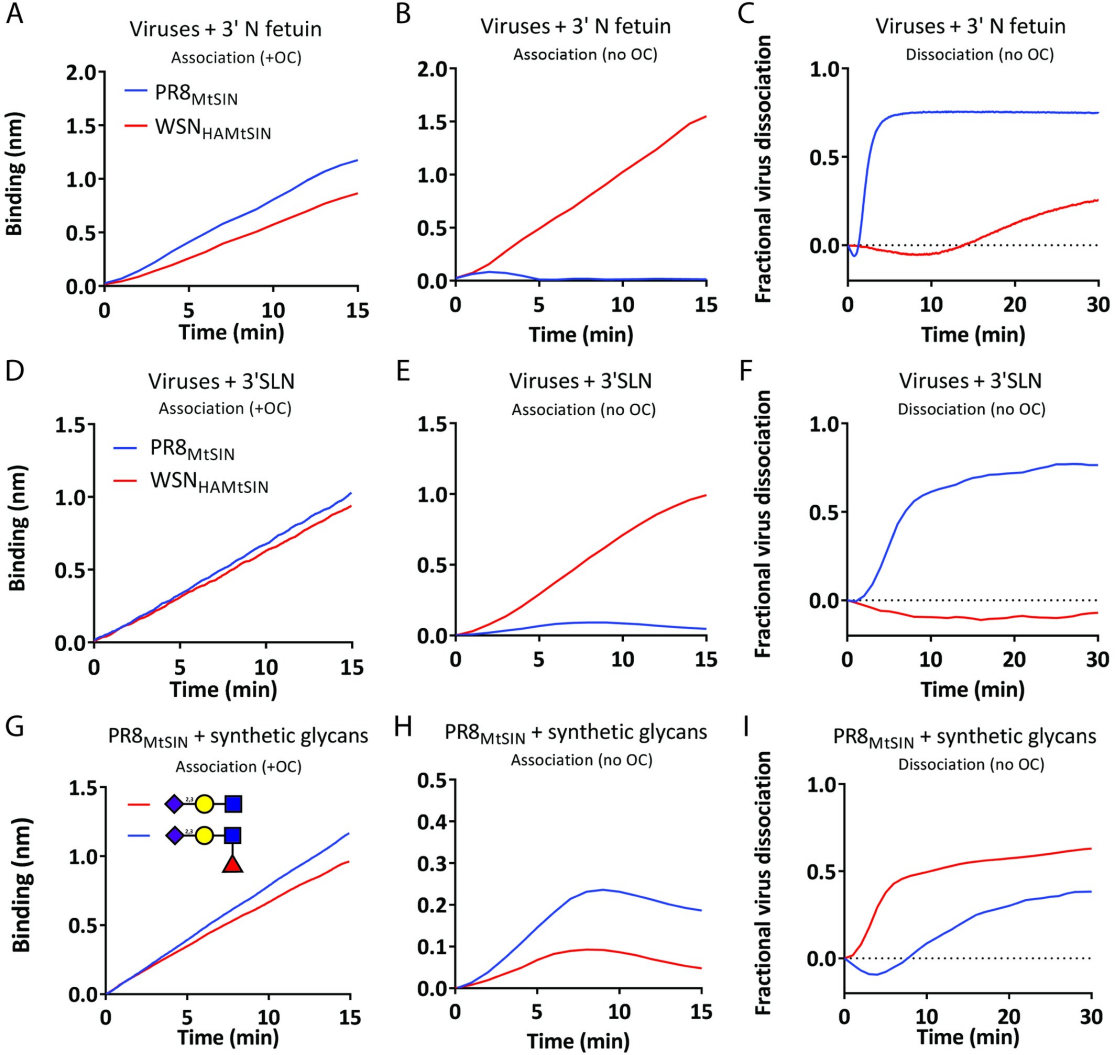
NA specificity affects the HA/NA balance. PR8_MtSIN_ and WSN_HAMtSIN_, both carrying the same HA_MtSIN_ but either NA_MtSIN_ (high NA activity) or NA_WSN_ (low NA activity), respectively, were bound at 30 pM concentration to biotinylated 3’SLN or Fc-tagged 3’N Fetuin loaded to maximum level in the presence (A, D) or absence (B, E) of 10 μM OC. Viruses bound to sensors in presence of OC (A, D) were washed three times in PBS and subsequently examined for NA activity-dependent self-elution in absence of OC (C, F). Virus dissociation in panel C and F is plotted on the positive Y-axis as fraction of virus released relative to the binding level reached in panel A and D respectively. Biotinylated 3’SLN or its fucosylated derivative (SLe^X^, NeuNAcα2,3Galβ1,4(Fucα1,3)GlcNAc) were loaded to maximum levels on SA sensors and bound with 30 pM PR8_MtSIN_ in the presence (G) or absence (H) of 10 μM OC for 15 minutes. After three washes in PBS the sensors loaded with virus in (G) were incubated in PBS without OC to allow determination of virus self-elution due to NA activity (I).

We determined the quantitative effect of NA activity on the dynamics of IAV-receptor interactions of two viruses carrying the same HA, but different NA proteins (WSN_HAMtSIN_ and PR8_MtSIN_; S2E Fig). Whereas the viruses displayed similar binding to both 3’N fetuin and 3’SLN when NA was inhibited (Fig 4A and 4D), only PR8_MtSIN_ binding was strongly reduced by its associated NA activity (Fig 4B and 4E). This observation corresponded well to the ~30-fold lower NA activity of WSN_HAMtSIN_ per virus particle (S7I Fig and S7J Fig). We conclude that, in addition to HA binding properties, NA activity drastically affects virus binding dynamics in the absence of NA inhibitors. The binding curves reflect the HA/NA balance which is quantifiable by empirical parameters like initial binding rate, the area under the curve (unit is min nm) and x,y coordinates of the peak (time and binding level).

NA activity-dependent self-elution of viruses bound in presence of OC provides quantification of other descriptive parameters that characterize the balance between HA, NA and receptor. Of note, the concentration of released particles is too low for their re-association to take place. As expected, PR8_MtSIN_ eluted much faster from 3’N fetuin or 3’SLN than WSN_HAMtSIN_ (which does not elute from 3’SLN). For both viruses the self-elution rate from 3’N fetuin was higher than from 3’SLN (Fig 4C and Fig 4F). This likely results from differences in specific activity of the NAs for these receptors but, as HA and NA are in competition for binding/cleavage of the same receptor, might also be affected by the K_D_ of monovalent HA-receptor interactions and thus be a reflection of HA/NA balance on a specific receptor. This possibility is further corroborated by an experiment where we compared the elution rates from 3’N fetuin and 6’N transferrin bt for two viruses (PR8_CAM2,6_ and WSN_WT_) carrying the same NA (from WSN_WT_) but a different HA (S8 Fig). Both viruses bind at similar, but relatively low, rate to 6’N transferrin bt (Fig 2E) whereas WSN_WT_ displays a ~3-fold faster binding rate to 3’N fetuin than PR8_CAM2,6_ (Fig 2D)_._ HA clearly affects the self-elution rate as, in combination with the same NA, self-elution from 3’N fetuin is much more efficient in companion of the weaker binding HA of PR8_CAM2,6_ (S8B Fig) than in companion of the stronger binding HA of WSN_WT_ (S8A Fig). Self-elution rates from 6’N transferrin bt are more similar for both viruses. Thus, whereas α2,3 SIA versus α2,6 SIA specificity of NA is seemingly opposite for PR8_CAM2,6_ and WSN_WT_, this is not caused by the NA itself (which is identical for both viruses) but by differences in their HAs.

Also a change of receptor (in this case to fucosylated 3’SLN, giving Sialyl-Lewis^X^; SLe^X^) was shown to have differential effect on the observed binding rate (Fig 4G) and NA-driven self-elution (Fig 4H and 4I). While *v*^*obs*^ is similar for SLe^X^ and 3’SLN in the absence of NA activity (Fig 4G), an active NA resulted in a lower *v*^*obs*^ and maximum binding level and a smaller area under the curve for 3’SLN (Fig 4H) and in a faster virion self-elution from 3’SLN (Fig 4I). Thus, receptor binding dynamics on 3’SLN and SLe^X^ differ due to a lower specific NA activity towards SLe^X^ which shifts the relative HA/NA balance on these receptors. In conclusion, BLI can be used to quantify changes in the dynamics of receptor binding due to an altered HA/NA/receptor balance. In Fig 4 we showed examples of the contribution of NA and receptor to this. Examples of HA effects on the balance are shown by comparing 4 viruses that only differ in their HA (S9 Fig). Clearly, the identity of the HA protein affects the initial binding rate as well as the area under the curve in the absence of NA inhibitor.

### Virus morphology is affected by the number of virus-receptor contacts

**Fig 5:**
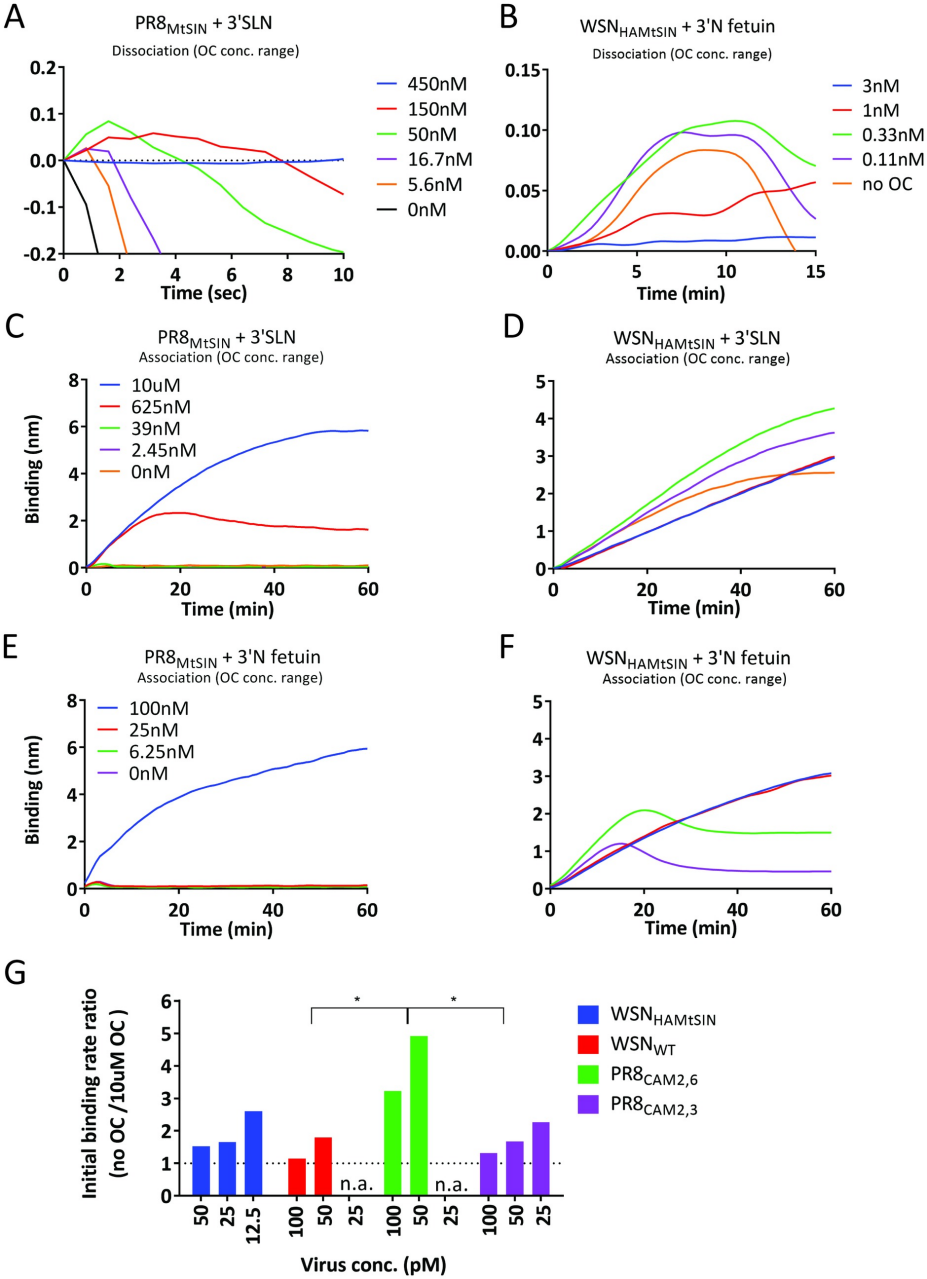
Contribution of NA to virion binding signal. (A-B) NA activity-dependent virus self-elution was examined for the first seconds (for PR8_MtSIN_; panel A) or minutes (for WSN_HAMtSIN_; panel B) of the dissociation phase. Viruses were loaded at 100 pM concentration to 3’SLN (PR8_MtSIN_) or 3’N Fetuin (WSN_HAMtSIN_) for 30 minutes in presence of 10 μM OC after which the sensors were washed in PBS and dissociation was examined at a range of OC concentrations as indicated in the panels. (C-F) PR8_MtSIN_ (C, E) and WSN_HAMtSIN_ (D, F), carrying the same HA_MtSIN_ but either NA_MtSIN_ (high NA activity) or NA_WSN_ (low NA activity), respectively, were bound at 100 pM concentration to biotinylated 3’SLN (C, D) or Fc-tagged 3’N Fetuin (E, F) loaded to maximum level. Binding was performed in absence (red lines) or in the presence of a range of OC concentrations as indicated in the panels. Significant differences between initial binding curves shown in panels C-F were analyzed by IBM SPSS statistic 24. In panel C, there was no significant difference between curves with 10μM OC and 625nM OC (P>0.1), whereas the curves of 10μM OC and 625nM OC were significantly different from 39nM, 2.45nM and 0nM (P<0.001). In panel D, the curves of 10μM OC and 625nM were significantly different from those of 39nM, 2.45nM and 0nM. There also was a significant difference between 39nM and 2.45nM. In panel E, the 100nM curves significantly differed from the other three OC concentrations. In panel F, there were significant differences between the 100nM (and 25nM) and 6.25nM and 0nM curves. (G) The ratio (initial virus binding rate *v*^*obs*^ in absence of OC)/(initial virus binding rate *v*^*obs*^ in presence of 10 μM OC) of four viruses carrying the WSN_WT_ NA in the background four different HAs was plotted (the individual binding curves are shown in S9 Fig). Standard deviations and significant differences between the mean initial binding rate ratios are indicated (* indicates P<0.05).

A small increase in reflection resulting in negative virus dissociation values, which cannot be explained by additional virus particle binding, was consistently observed during the initial phase of self-elution (e.g. Fig 4C, 4F and 4I). The effect was most prominent when NA activity was low. The possible role of NA activity herein was further examined by performing self-elution of PR8_MtSIN_ from 3’SLN and WSN_HAMtSIN_ from 3’N fetuin in the presence of an oseltamivir concentration range (Fig 5A and 5B). Duration and magnitude (maximally ~0.12nm) of the increase in the apparent binding level, which was not observed when NA was fully inhibited, depended on the degree of NA activity. The effect must reflect a neuraminidase activity-dependent change in the receptor-bound virus particles as free virus particles that can bind are absent. Cleavage of SIAs by NA is expected to gradually decrease the number of HA-SIA contacts between receptor-coated surface and virus until the particle dissociates. Relaxation of virus particle binding (by less contacts) could result in an altered binding conformation of a particle that, at the maximal number of contacts, might be tightly squeezed against the receptor surface. Such a morphological change likely explains the observed, NA-activity dependent, changes in reflection.

### A contribution of NA to receptor binding, correlating to its level of activity, can be quantified by BLI

Remarkably, the initial binding rate of WSN_HAMtSIN_ to 3’N fetuin was higher in absence than in presence of a high concentration of OC (compare Fig 4A and 4B). Several avian NA genotypes have been shown to possess a 2^nd^ SIA-binding site, alternatively referred to as hemadsorption site [44,45], but such a site is not conserved in WSN NA and OC binding to this site has never been demonstrated. However, active site mutations that abolish catalytic NA activity have resulted in NA-dependent hemagglutination, which could be inhibited by OC [46,47]. We therefore examined the effect of OC on binding of WSN_HAMtSIN_ (low NA activity) and PR8_MtSIN_ (high NA activity) virions to 3’SLN and 3’N fetuin. Complete NA inhibition gave binding curves displaying a continuous increase of virus binding (Fig 5C–5F, blue lines). However, the *v*^*obs*^ of WSN_HAMtSIN_ increased at lower OC concentrations in a concentration-dependent way (Fig 5D and Fig 5F). In time, the curves bent down by depletion of SIA receptors due to NA activity. In contrast, the *v*^*obs*^ of PR8_MtSIN_, which has a highly active NA in comparison to WSN_HAMtSIN_, directly decreased strongly at lower concentrations of OC (Fig 5C and Fig 5E). The results imply that NAs with a relatively low cleavage rate (low *k*_*cat*_) contribute to the *v*^*obs*^ by binding with their active site to the SIA receptor. To strengthen this conclusion, we quantified the enhancement of the initial binding rate by WSN_WT_ viruses carrying the same NA but four different HAs (Fig 5G and S9 Fig). Whereas all four viruses displayed an effect of NA on *v*^*obs*^, the virus with the lowest HA-dependent initial binding rate (PR8_CAM2,6_), was relatively most enhanced in initial binding rate by NA-dependent binding (S9E Fig and S9F Fig). This demonstrates that the degree of the contribution of NA to *v*^*obs*^ is influenced by its HA partner. In conclusion, the NA protein contributes to the initial binding rate balance by binding with its active site to SIA receptors and the magnitude of the effect is probably influenced by the HA/NA balance.

### NA activity enables virus rolling over a receptor-coated surface

**Fig 6:**
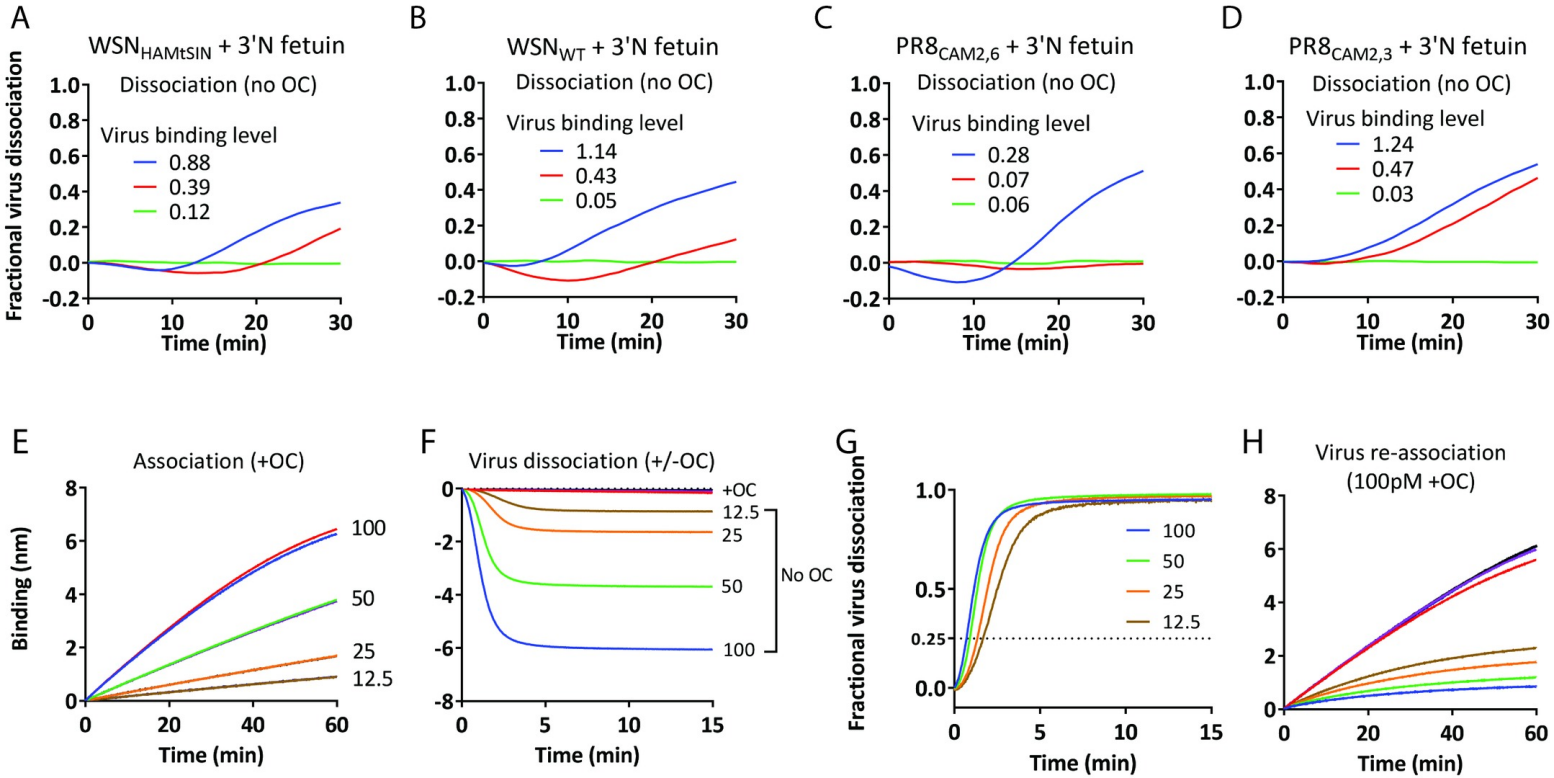
Viruses are rolling over the receptor surface before NA activity-driven dissociation takes place. (A-D) Virus dissociation of the indicated viruses in the absence of OC is plotted on the positive Y-axis as fraction of virus released relative to the binding level reached in the presence of OC (indicated in nm and shown in S9 Fig). (E) PR8_MtSIN_ was bound in duplicate at four virus concentrations to eight SA sensors containing biotinylated 3’N+O fetuin bt in presence of 10 μM OC. (F) Viruses bound to the sensors in (E) were allowed to dissociate at each concentration in absence or presence of OC for 15 minutes. (G) Virus dissociation in panel F is plotted on the positive Y-axis as fraction of virus released relative to the binding level reached in panel E. (H) After the dissociation step (F), sensors were regenerated at pH2, thereby removing all bound viruses but leaving the 3’N+O fetuin bt bound to the sensor. All regenerated sensors were subsequently allowed to re-bind PR8_MtSIN_ at a concentration of 100 pM to determine the degree of de-sialylation that occurred in (F).

Fig 6A–6D show NA-dependent dissociation (self-elution) of the viruses used above (Fig 5G and S9 Fig), carrying different HAs and the low activity NA of WSN. All four viruses showed faster dissociation when more virus was loaded, indicating that there is positive cooperativity between viruses in respect to self-elution rate. The only plausible mechanism by which viruses can assist each other in self-elution is by exerting NA activity on a larger surface area than their own contact area, implicating that virus particles move over the receptor-coated surface. To test this hypothesis a concentration series (12.5 pM to 100 pM) of PR8_MtSIN,_ harboring a highly active NA, was bound to 3’N fetuin in presence of OC resulting in virus saturation levels on the sensor surface ranging from ~8% to ~ 60% (Fig 6E), based on a maximal binding level of 9 nm after prolonged binding (S4A Fig). Subsequent NA-dependent self-elution (Fig 6F) confirmed that higher virus loading levels result in faster self-elution as shown by plotting fractional virus dissociation against time (Fig 6G). We next determined whether virus particles are indeed able to clear SIAs from a larger surface area than their own contact area during self-elution. After self-elution, sensors were regenerated and re-bound with a high concentration (100 pM) of virus (Fig 6H). Clearly, even at a level where only ~8% of the surface was bound with virus, self-elution created a surface to which only very limited re-binding of virus particles could take place indicating extensive removal of SIAs from the complete surface. As the concentration of virus released from the surface is too low for re-association and non-bound virus does not contribute to receptor cleavage, we conclude that virus particles exert NA activity while moving over the receptor-coated surface.

**Fig 7:**
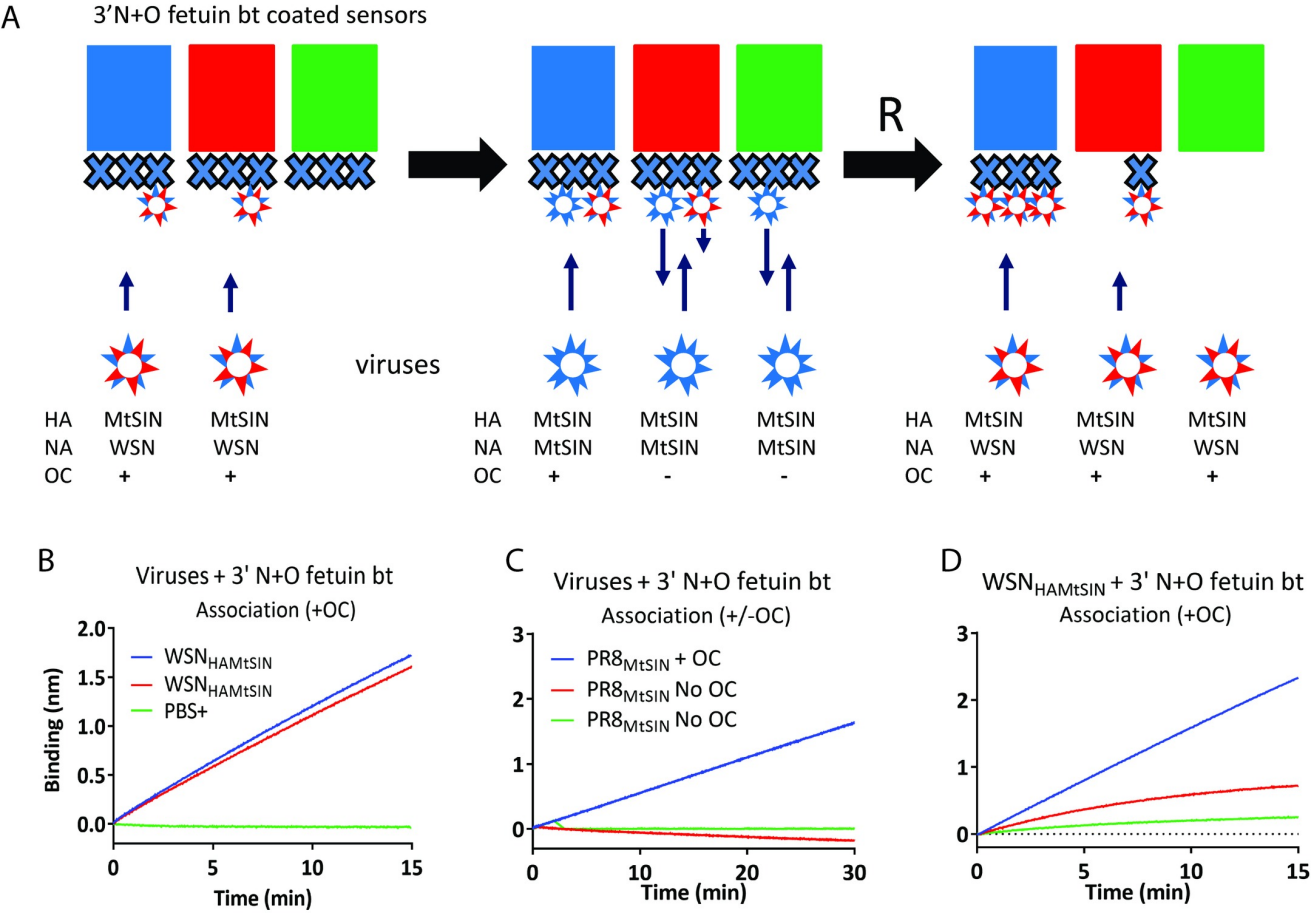
Virus rolling is driven by NA activity. (A) Schematic representation of the experimental set-ups. Colours of the sensors correspond with the colours of the lines in graphs B-D. (A). Biotinylated 3’N+O fetuin (3’N+O fetuin bt) is indicated by the large Xs. Origin of the HA and NA proteins of the viruses used is indicated, as well as the absence or presence of OC during the incubation of the sensors with the viruses. The left, middle and right panels correspond with the graphs shown in B,C, and D (A). The capital R indicates the regeneration of the sensors. (B) 75 pM WSN_HAMtSIN_ (with low NA activity) was bound in presence of 10 μM OC to two sensors containing biotinylated 3’N+O fetuin (3’N+O fetuin bt) (loaded to maximum level) for 15 minutes reaching ~15% of the maximum binding level. A control sensor was dipped in PBS. (C) The sensors from B were allowed to bind 30 pM PR8_MtSIN_, in the presence or absence of 10 μM OC. (D) Regenerated sensors were allowed to re-bind WSN_HAMtSIN_ at a concentration of 100 pM to determine the degree of de-sialylation that occurred in (C).

Migration of attached IAV particles over a receptor surface necessarily depends on the very high K_D_ of monovalent HA-SIA interactions resulting in their rapid formation and dissociation. We hypothesize that NA, in combination with the highly dynamic formation and release of individual HA-SIA interactions, drives virus rolling by the generation of a receptor gradient due to its receptor destroying activity. Rolling of virus particles is predicted to be faster for virus particles with higher NA activity. This was tested by comparing the binding and dissociation dynamics of PR8_MtSIN_ and WSN_HAMtSIN_, which have the same HA but have high and low NA activity, respectively. The experimental set-up shown in Fig 7A links sensors to BLI graphs by color coding. First (Fig 7B), biotinylated 3’N+O fetuin -coated sensors were loaded with WSN_HAMtSIN_ to a ~15% saturation level in the presence of OC (blue and red) or incubated in buffer (green). In the next step (Fig 7C), these sensors were incubated with PR8_MtSIN_ in the presence or in the absence of OC. In presence of OC (blue) efficient binding of PR8_MtSIN_ to the large virus-free area takes place. In the absence of OC, high NA activity prevents long lasting binding of PR8_MtSIN_ to an empty sensor (green). The slightly negative slope of the WSN_HAMtSIN_ loaded sensor (red) represents minor dissociation of WSN_HAMtSIN_ in absence of OC, as expected on basis of its low NA activity. This also implicates that WSN_HAMtSIN_ elution is not appreciably assisted by PR8_MtSIN_. In the last step of the experiment (Fig 7D), the sensors were regenerated to remove all bound viruses followed by probing the residual SIA content by its association capacity with WSN_HAMtSIN_ in the presence of OC. As expected, the blue sensor, to which both viruses were bound in presence of OC, was as efficiently bound by WSN_HAMtSIN_ as in the first round. Also as expected, the green sensor to which no virus was bound in the first step and PR8_MtSIN_ in absence of OC in the second step was efficiently cleared from SIAs, as demonstrated by its poor capacity to re-bind to WSN_HAMtSIN_. In contrast, binding of WSN_HAMtSIN_ to ~15% of sensor surface in the first step (red) prevented complete removal of SIAs by PR8_MtSIN_ in the second step as demonstrated by considerable re-binding of WSN_HAMtSIN_ in the third step (red). This implies that WSN_HAMtSIN_, by marginal rolling over the surface because of its low NA activity, protects its contact surface with the sensor against the NA activity of PR8_MtSIN_, which cleaves SIAs of all the non-protected surface area. We conclude that NA activity is the driver of virus rolling on a receptor-coated surface.

## Discussion

The *ménage a trois* between IAV HA, NA and (decoy) receptors largely determines viral fitness and host cell tropism. Here, by studying their highly dynamic but poorly understood interplay using BLI, we obtained novel mechanistic and quantitative insights into IAV-host interactions.

### A model for IAV-receptor surface interaction

**Fig 8:**
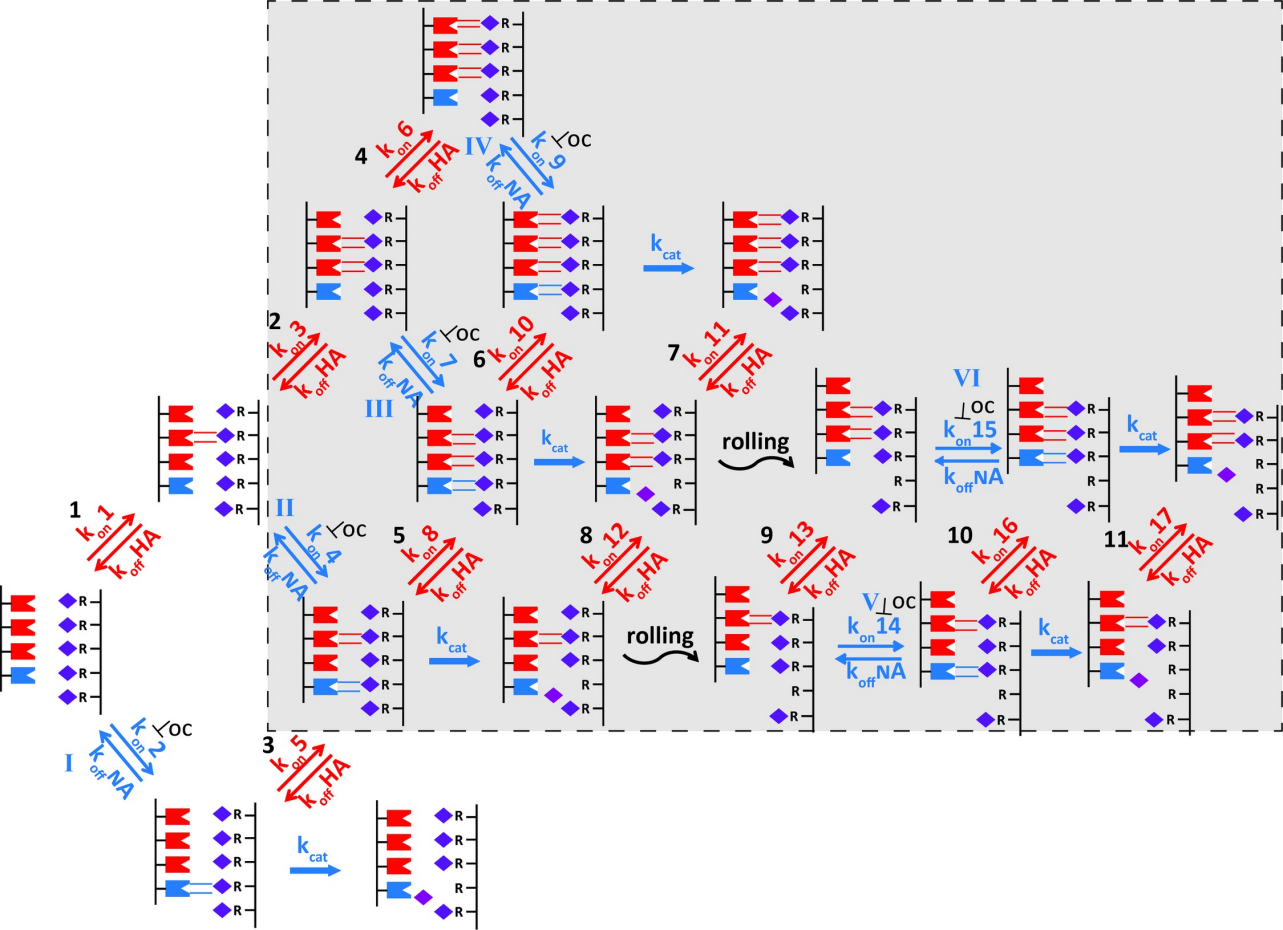
Kinetic model showing the basic flow of events taking place during virus particle binding. Schematic representation of virus binding to a receptor-coated surface. Part of the viral envelope, containing HA (red symbol) and NA (blue symbol), and the sensor surface, coated with SIA (purple diamond)-containing receptors (R), is shown. Kinetically different steps lead to multivalent interaction between IAV and a receptor-coated surface. The initial HA-dependent binding event (step 1 in red) is a virus concentration-dependent intermolecular process governed by a binding rate constant *k*_on_ (with the unit M^−1^ s^−1^) and a dissociation constant *k*_off_ (with the unit s^−1^). Subsequent HA binding steps (e.g. steps 2 and 4) are intramolecular with a *k*_on_ (not necessarily the same as in the first step) and *k*_off_ (both with the unit s^−1^). For IAV, having a K_D_ (k_off_/k_on_) of ~0.3 to ~3mM for a monovalent HA-receptor interaction, binding at pM concentrations inevitably results in a low binding rate that is mostly determined by the first binding event. NA can also contribute to the initial binding rate. This contributory effect can be inhibited by OC and is therefore attributed to binding of receptor to a NA catalytic site. The contribution of NA to receptor binding is determined by a dissociation constant (*K*_*D*_ = *k*_*off*_*/k*_*off*_) for the substrate (step I in blue) and a catalytic rate constant (*k*_*cat*_; bold blue arrow) determining the receptor cleavage rate. A lower *k*_*cat*_ will result in prolonged receptor binding before cleavage or dissociation takes place. This will enhance the chance for additional binding events (mostly by HA, which is present at higher density than NA), thereby promoting the cascade of multivalent interactions responsible for tight virus binding. Given the lower K_D_ (30μM~600μM) [51] of NA, in comparison to HA, for interaction with sialosides, a considerable contribution to the initial binding rate by NA is expected even whereas the NA/HA ratio of a virus particle is generally quite low. Longer lasting, BLI-detectable, binding requires the formation of additional HA- and/or NA–SIA bonds, which is indicated by the grey shaded area. Initial binding events will be hardly detected due to the low levels of equilibrium binding in step 1 and I. During the BLI-detectable phase of binding, HA- and NA–SIA interactions are formed and broken in a virus concentration independent mode with the result that all binding states can rapidly interconvert via binding/dissociation events 2 to 11 and II to IV. The number of simultaneous interactions that can occur is logically dependent on receptor density and k_off_/k_on_ ratios but how many simultaneous interactions suffice to keep a virus particle bound to the surface remains unknown. The experiments shown in Fig 1 suggest the number of interactions required is very low. Theoretically, the dynamics of HA-SIA interactions allow a virus to roll over the surface but experiments shown in Fig 7 show that NA activity strongly stimulates rolling (and eventually leads to virus dissociation). This is schematically indicated by the curled arrows where NA cleavage activity creates receptor-free positions on the surface. The receptor gradient caused in this way is probably the driving force for virus rolling but the direction in which the virus rolls (away from the empty position or “reaching over” the empty position) still needs further research.

We combined the key findings into a schematic model (Fig 8). The initial monovalent HA-SIA interaction is virus concentration-dependent and governed by a binding rate constant *k*_on_ (M^−1^ s^−1^) and a dissociation constant *k*_off_ (s^−1^). Its high *K*_*D*_ (*k*_on_/*k*_off_ ≈ 0.3 to ~3mM [48–50] cannot be easily determined and makes monovalent virus-receptor interactions undetectable by BLI too. In combination with picomolar virus concentrations, this high *K*_*D*_ inevitably results in the observed low virus binding rate. Subsequent HA binding steps are intramolecular (determined by a different *k*_on_ and *k*_off_ with the unit s^−1^), resulting in multivalent binding. Remarkably, and counterintuitive to its receptor destroying activity, NA can contribute to the initial binding rate. Receptor residence time in the NA substrate binding site is determined by the binding constant (*K*_*D*_ = *k*_*on*_*/k*_*off*_, range is 30μM~600μM [51] and catalytic rate constant (*k*_*cat*_). Obviously, a low *k*_*cat*_ promotes the chance on secondary binding events (mostly of HA), which will also be affected by the NA/HA virus incorporation ratio (S7K Fig). BLI-detectable virus binding is, due to multivalency, virtually irreversible but highly dynamic as individual HA- and NA–SIA interactions are rapidly formed and broken in a virus concentration independent mode. The number of simultaneous interactions required for virtually irreversible (*k*_off ≈_ 0) binding is low and logically depends on receptor density and the k_off_ and k_on_ values of a virus (Fig 1E). The rapid interconversion of binding states via the association/dissociation events shown in Fig 8 enables a virus to roll over the surface. NA activity (curled arrows) is required to drive rolling, most likely by creating a receptor gradient that forces a virus to roll away from the empty receptor positions. Receptor cleavage eventually results in virus dissociation when receptor density becomes too low to support tight multivalent binding. This model clearly sketches important biological as well experimental consequences.

### Equilibrium binding models are poorly applicable to IAV-receptor interaction

Quantification of IAV-receptor binding usually focuses on determination of the dissociation constant *K*_*D*_. However, equilibrium binding models did not apply, even at low receptor densities, and binding curves were dominated by concentration-independent avidity effects resulting in virtually irreversible binding. The binding curves are in agreement with a random sequential adsorption model [52,53]. In such models irreversible particle binding proceeds to a plateau at ~55% occupation of the binding surface (the jamming limit). However, IAV particle binding could slowly proceed to complete saturation of the surface (Fig 1 and S4A Fig). We attribute this to virus rolling over the surface, a mechanism by which at an eventually very low rate sufficient space for new binding events is created. Current, inherently complex, models for polyvalent binding lack general applicability [54,55] and cannot be used to determine the kinetic constants of the different binding events shown in Fig 8. An apparent *K*_D_ for IAV-receptor interaction has been determined [35] from fractional saturation (f) levels obtained after 60 min at different receptor densities (*K*_D_ = ([virus](1-f))/f). Such an application of BLI as an endpoint binding assay (shown for our data in S4F Fig) falsely assumes an equilibrium binding model and further errors into apparent *K*_*D*_ determinations are introduced by low binding rates (as observed for weak binders or at low virus concentration, S4A Fig and S4B Fig) that prevent reaching a binding plateau within 60 min.

### The initial binding rate is the relevant quantifiable parameter for IAV binding

IAV binding to cells is poorly reversible [56] and adherence of only a few particles is already sufficient for productive cell infection. Thus, where equilibrium binding levels or binding to saturation are not an issue, the initial binding rate *v*^*obs*^ is a physiologically relevant parameter for IAV binding. It can be determined by BLI, but not easily by endpoint assays like conventional glycan arrays, more recently developed shotgun glycan arrays [57] or receptor-coated 96-well plate based assays. Direct proportionality between virus concentration and *v*^*obs*^ during the initial binding phase allows quantitative comparison of viruses of unknown concentration by determination of the relative binding rate constants for different receptor pairs. Coating of sensors with recombinant glycoproteins, in a homogeneous orientation by virtue of their N-terminal tag (e.g. Fc-tag, biotinylated Bap-tag), provides analysis of receptor surfaces on which the sialoglycans receptors are attached in protein-linked conformations as encountered *in vivo*. Genetic engineering enables binding studies to glycoproteins carrying specific glycan-types (N-linked, O-linked) or glycan-density whereas further tuning of glycan structure can be accomplished by glycoprotein expression in cell lines with specific differences in their glycosyltransferase expression patterns. This will enable a much more refined analysis than the often used biotinylated polyacrylamide molecules carrying randomly distributed glycans and supposed to adopt a spherical configuration with a ~15nm diameter [58].

### Contribution of NA to virion binding

The recent emergence of H3N2 strains displaying NA-dependent hemagglutination hint at a role for NA in determining the changes in receptor binding that accompany and/or drive virus evolution. This phenomenon is thought to reflect the gained capacity of NA to bind receptors that are refractory to cleavage by NA [46,59,60]. By using highly sensitive BLI assays we showed that the NA of strain WSN_WT_ contributes to the initial binding rate even though it is capable of receptor cleavage leading to virus elution. This effect is exerted by binding of the receptor to the NA catalytic site, as demonstrated by the inhibitory effect of OC, and is negatively correlated with the specific activity of the NA.

Changes in HA-receptor binding specificity and avidity are thought to be prime factors in causing, or responding to, antigenic change [61,62] or infection of an altered host cell range. Secondary changes in NA have been proposed to restore a critical HA/NA functional balance. Now, considering the evidence for a role of NA in receptor binding, more complex scenarios should be considered. For instance, changes in NA might facilitate (transient) functional changes in HA by (temporarily) contributing to the initial virus binding rate. BLI allows quantification of such effects by determination of the relative increase by NA of HA-dependent virion binding (Fig 5 and S9 Fig).

### NA activity-driven virus rolling and the role of the HA/NA balance

A functional HA/NA balance has primarily been described by weighing separately determined HA binding and NA activity properties, using isolated proteins or virions. BLI enables quantification of the separate contributions of HA and NA to virus binding rates. The strength of BLI lays in studying the simultaneous effect of NA and HA, and thereby their balance, on the dynamics of virus-receptor interactions. Virtually irreversible binding is the result of multiple HA-SIA interactions that rapidly associate and dissociate, thereby providing access for NA to temporarily unbound SIAs. SIA cleavage by NA creates a receptor density gradient that drives virus rolling, most likely away from the receptor-free spot in the direction of higher receptor density, or alternatively, by taking “a large step” to a site beyond the de-sialylated receptor. Ultimately, when receptor density becomes too low, virus dissociation will occur. The resulting binding/dissociation profiles can be recorded by BLI and are a quantitative reflection of the HA/NA balance. Yet lacking a mathematical model, these profiles can be described by empirical parameters (initial binding rate, area under the curve and x,y coordinates of the peak).

Kinetic binding rate constants for HA and NA (*k*_*on*_ and *k*_*off*_) and the NA catalytic rate constant (*k*_*cat*_) will determine virion rolling characteristics (Fig 8). In addition, the HA/NA ratio and distribution pattern in the virus envelope will determine the frequency at which NA will be present in the contact area between virus and receptor surface where receptor cleavage can take place. As such, HA/NA ratio and distribution pattern are additional variables that can be involved in functional evolution of the HA/NA balance and its effect on virus rolling. Whereas mostly a random distribution of HA over the viral envelope has been observed, the less abundant NA has been shown to occur as singular tetramers as well as small clusters thereof. The latter has mostly been observed for IAVs with a filamentous morphology [63–65]. Whereas IAVs harvested from cell culture usually display a spherical (~100 to 120 nm diameter) or slightly pleomorphic shape, filamentous morphology is frequently, but not always, observed in clinical samples (reviewed in [65,66]) although the loss of a filamentous phenotype is not absolutely required for adaptation to growth in cell culture. Filamentous IAVs usually have a diameter of 80 to 100 nm and a length up to 20 μm. Patches of NA clusters at the tip and the base of long filaments have been described [63,64] but other reports suggest the presence of NA along the length of the filament [67]. Several functions for a filamentous morphology have been proposed (reviewed in [65,66]) including a role in clearance of SIAs from mucus by long budding filaments that have not pinched of from the cell surface [68]. Results obtained in this study are confined to the behavior of spherical IAVs and additional studies are required to see if filamentous IAVs can move over a surface by lateral rolling, crawling or a caterpillar-like motion. Interestingly, unidirectional motility of a filamentous influenza C virus over a receptor-coated glass-slide was recently demonstrated by microscopy [69] where as a more random movement of spherical IAV particles was observed on fetuin-coated glass slides by total internal reflection microscopy. Directional movement of virus particles at two different velocities, both dependent on NA activity, was reported [69]. Earlier, the combination of a receptor-binding and a receptor-destroying enzyme has been proposed to enable IAV movement over a cell surface by a mechanism of repeated cycles of receptor binding, receptor release and receptor cleavage [70].

Spherical and filamentous IAV particles probably both occur *in vivo* and may very well have different functions. Whereas NA-dependent motility of filamentous particles through a mucus layer might be difficult, they might be better suited for spreading the infection to neighboring cells or clearing the mucus layer from their SIA content. Spherical IAV particles on the other hand, are likely obtained during *in vivo* infection of humans by inhalation of aerosols containing a relatively low number of virions that somewhere get stranded on the mucus layer covering epithelial cells of the respiratory tract. Thus, whereas quantitative measurements of spherical IAV binding kinetics by BLI should yield valuable data for modeling and testing the movement of such particles through a mucus layer and over epithelial cell surfaces, additional experiments are required to determine whether this can be extended to filamentous IAV particles.

IAV was shown to require NA activity for penetrating through a mucus layer *in vitro*[24]. The irreversible but highly dynamic binding mode that leads to virus rolling results in virus self-elution from a receptor-coated surface only upon efficient clearance of receptors from the complete surface. Considering the low amount of virions confronted by a large amount of mucus and epithelial cells, we propose that virions, once attached to the mucus, remain receptor bound for their entire extracellular life. Soluble, densely sialylated mucins in the mucus layer (2–20 μm thickness) form, by polymerization, a mesh-like structure with an average pore-size of ~500nm [71] through which the virions need to move to reach the pericilliary layer. In this layer, the cilia of columnar epithelial cells (0.2 to 0.3 μm in diameter; 6 to 7 μm in length) are covered by membrane-spanning mucins and tethered muco-polysaccharides that protrude into the narrow (~200nm) space between cilia from which soluble mucins or 40 nm beads were shown to be excluded [72]. We consider it likely that rolling is necessary for gaining the directionality and even traction required for efficiently penetrating this heavily sialylated maze to reach sites on the epithelial cell membrane that permit virus entry. Whether, and to which extent, rolling also takes place over the epithelial cell surface in order to arrive at a site that permits entry by endocytosis remains an open question. The overall density of SIAs on the cell surface is estimated to be high [73,74], but also very heterogeneous. IAV entry by clathrin-mediated endocytosis was shown to take place by de novo formation of clathrin-coated pits at sites where a virus particle was bound [75]. Thus, it seems likely that in case of rolling the virus becomes arrested at a specific place in time. Tight binding to specific receptors might be required for this but these have so far not been identified. Note that the virus may also surf together with a tightly-bound glycoprotein over the cell surface. Rolling might also be important for cell-to-cell virus spread. Virions budding from de-sialylated cells, resulting from NA activity, may even utilize sialylated mucins covering the epithelial cell layer as a rolling track to neighboring cells. In this perspective, an optimal HA/NA balance should be considered as the combination of HA and NA kinetic parameters, including factors like HA/NA ratio and distribution, that optimally supports virion rolling over distinct surfaces coated with a diversity of receptors. Also protective antibodies targeting IAV receptor binding sites will function within this context with an important role for the HA/NA/receptor balance as they will compete with receptors for interaction with IAV [61,62]. The BLI-based methodology that we have established here is well-suited to quantify such processes using a highly versatile, modifiable and controllable receptor repertoire.

## Methods

### Generation of recombinant viruses and virus cultivation

Influenza A/WSN/33 (H1N1) (WSN_WT_) and reassortant virus strains WSN_HAMtSIN_, PR8_CAM2,6_, PR8_CAM2,3_ were made by standard reverse genetic procedures [76] creating viruses with different HA-encoding genome segments (from WSN [Accession No. P03454.1], PR8 Mount Sinai [Accession No. ADX99484.1], PR8 Cambridge [Accession No. NP_040980.1], and PR8 Cambridge containing E190D introduced by sited-directed mutagenesis according standard procedures) in the background of the other seven WSN genome segments (Taxonomy ID: 382835)(see S2 Fig for a schematic outline). Influenza A/Puerto Rico/8/34/Mount Sinai (PR8_MtSIN_; Taxonomy ID: 183764) and reassortant virus strain TX77_NAMtSIN_ (containing the HA gene of A/Bilthoven/1761/76 [Accession no. AY661006.1]) in the background of PR8_MtSIN_ [77] as well as all other viruses were grown in MDCK-II cells as described previously [78] and stored at −80°C. Virus titers were determined by measuring the TCID50 on MDCK-II cells. Sequences of the HA and NA genes were confirmed by sequence analysis (Macrogen).

### Virus particle, HA and NA quantification

For quantification, virus samples were concentrated by TCA precipitation [79] and applied to standard 10% SDS-PAGE gels for separation of viral proteins followed by silver staining. Silver-stained polymerase bands were quantified by densitometry on silver staining gels as outlined and shown in supplementary S7A Fig, S7B Fig and S7C Fig. HA and NA amounts were quantified by western blotting of standard 10% SDS/PAGE gels. Monoclonal antibodies used for detection and quantification by densitometry were FI6 (for quantification of HA) [80], N1-7D3 (for quantification of PR8_MtSIN_ N1) [81] and GT288 (for quantification of WSN N1). Recombinant purified HA and NA proteins (see below) were used for standard curves (S7D–S7I Fig).

### Electron microscopic analysis of virus particles

Before the application of samples, 400-mesh copper grids with a pure carbon film were exposed to a glow discharge in air for 20 s to make them hydrophilic. Ten microliters of the virus preparations was applied to the grids and incubated for 2 min. Excess sample was blotted with a filter paper. For negative staining, 10 μL of 2% phosphotungstic acid at pH 6.8 was applied. After 1 min, excess stain was blotted and grids were left to dry. The specimens were examined in a JEOL JEM1400 transmission electron microscope at 120 kV and images were taken at a magnification of 30.000x with a Matataki 2K x 2K camera.

### Construction of expression vectors

Human codon-optimized H1-encoding and N1-encoding cDNAs of PR8_MtSIN_ and WSN_WT_ (Accession no. P03454.1 for WSN HA, ACF54601.1 for WSN NA, ADX99484.1 for PR8 HA, and P03468.2 for PR8 NA) were cloned in pCD5 (HA) or pFRT (NA) expression vectors flanked by CD5 signal peptide-, GCN4-isoleucine-zipper trimerization (for HA) or tetramerization (for NA) domains, and Strep-tag II-encoding sequences similarly as described previously [16,82]. Codon-optimized cDNA fragments encoding the variable domains of the heavy and light chains of antibody FI6 [80] were synthesized by GenScript USA Inc and cloned in-frame into pCAGGS vectors containing human IgG1 heavy and light constant domains, respectively, similarly as described previously [83]. Codon-optimized bos taurus fetuin-encoding cDNA (Accession no. NP_776409.1) was cloned in the pCAGGs expression vector fused in frame to sequences encoding a human Fc-tag with or without a tandem repeat of the 15-amino-acid biotin acceptor peptide (Bap)-tag [84]. Biotinylated fetuin is referred to as fetuin bt. Mutations for knocking out O-linked glycosylation sites were introduced into the fetuin gene by using the Q5 site-directed mutagenesis kit (NEB) and confirmed by sequencing. Human codon-optimized cDNA encoding biotin protein ligase (BirA)[84] was cloned in pCD5 in frame with sequences encoding a CD5 signal peptide and a 6His tag.

### Protein expression and purification

Expression vectors were transfected into HEK293T (ATCC CRL-3216),CHO K1 (ATCC CCL-61) and GnTI-deficient CHO 15B cells [85](from Ineke Braakman, Utrecht University, the Netherlands) using polyethyleneimine I (PEI) similarly as described previously [16,82]. CHO cells are deficient in α2,6 sialyltransferases and therefore exclusively synthesize 2,3 SIA linkages [86,87]. Tissue culture supernatants were harvested 5–6 days post transfection. Recombinant HA and NA proteins were purified using Strep-Tactin sepharose beads according to the manufacturer's instructions (IBA, Germany). Fc tag-containing proteins were purified using protein A sepharose beads (GE Healthcare), similarly as described previously [83]. For an overview of the different fetuin constructs see S2C Fig. The concentration of purified protein was determined by using a Nanodrop 1000 spectrophotometer (Isogen Life Sciences) according to the manufacturer’s instructions and analyzed by 10% SDS-PAGE followed by visualization of protein bands using a Colloidal Blue Staining kit (Invitrogen). Biotinylated transferrin (Sigma) contains two glycan chains exclusively carrying α2,6 SIAs [88,89](referred to as 6’N transferrin bt).

### Neuraminidase enzyme activity assay

The activity of IAV virus particles as well as recombinant soluble NAs was determined by using a fluorimetric assay similarly as described previously [16]. In short, viruses and NA preparations were subjected to 2-fold serial dilutions in reaction buffer (50 mM Tris-HCl, 4 mM CaCl2, pH 6.0) in a flat-bottom 96-well black plate (Greiner Bio-One). Subsequently, a similar volume of reaction buffer containing 200 μM 2′-(4-methylumbelliferyl)-α-d-N-acetylneuraminic acid (MUNANA; Sigma) was added to each well, mixed well, and incubated at 37°C for 60 min. The reaction was terminated with the stop solution (0.1 M glycine, 25% ethanol, pH 10.7). The fluorescence of the 4-MU reaction product was immediately determined in relative fluorescence units (RFUs) using a Fluostar Optima plate reader (BMG Labtech, Mornington, Australia) with excitation and emission wavelengths at 340 and 490 nm, respectively.

### Glycan array assay

Microarrays were printed as described previously [90]. The glycan array analysis of the HA proteins was performed as previously described [91]. Briefly, 200 μg/ml recombinant HA was precomplexed with a horseradish peroxidase-linked anti-streptavidin tag antibody and an Alexa Fluor 488 anti-mouse antibody (4:2:1 molar ratio) for 30 min at 0°C, prior to incubation for 90 min on the microarray slide under a microscope cover glass in a humidified chamber at room temperature. After repeated washes with phosphate-buffered saline (PBS) with 0.05% Tween, PBS, and deionized water, the slides were immediately subjected to imaging.

### Biolayer interferometry

BLI analysis was performed on an Octet QK machine using standard streptavidin (SA) or protein A bio-sensors. PBS with calcium and magnesium (PBS+/+) was used as standard assay buffer. Receptor loading was performed by loading biotinylated receptors (synthetic glycans or proteins) to SA sensors or Fc-tagged glycoprotein receptors to protein A sensors. Unless otherwise specified, sensor were loaded with receptor to maximum levels (no further increase in reflection) using 100 nM synthetic glycan or 4 μg/ml glycoprotein as loading sample concentration. After loading sensors were washed in PBS+/+ until a stable baseline was obtained. Virus binding was performed by moving receptor-loaded sensors to wells containing 100 μl virus sample at the indicated concentrations (the use and concentration of OC is indicated where applicable). Then virus-loaded sensors are usually moved to PBS+/+ in presence of 10 μM OC to examine virus dissociation (consistently producing a flat line), or washed 3 times for 3 seconds in PBS+/+ to remove OC and next transferred to PBS+/+ without OC to measure NA activity-driven self-elution. Alternatively, in order to determine simultaneous action of HA and NA virus binding was analyzed from the start in PBS+/+ in the absence of OC. Regeneration of sensors, preserving the binding of biotinylated receptors but removing all bound virus, was performed by dipping sensors briefly in 10 mM Tris/Glycine buffer pH 2. PR8_MtSIN_-specific antibody (03/242 from NIBSC) was used for detection of virus binding in presence of OC with or without prior sensor generation. Fetuin-coated sensors were also analyzed for their lectin-binding properties. To this end, fetuin-coated sensors were incubated with different lectins (80ng/ul; SNA, MAL-I, MAL-II, ECA, all from Vector Labs) and lectin-binding curves were obtained.

### Software and Statistical analysis

Each BLI experiment was repeated at least twice. Representative experiments were graphed. Initial binding rates, corresponding to the sloops of the binding curves during the first few minutes of the virus-binding experiments, were determined by second order polynominal equation (GraphPad Prism 7.04). The correlation between virus particle numbers and the initial binding rate was determined by linear regression and Pearson r analysis using GraphPad Prism 7.04 software. Significant differences between curves were analyzed by univariate analysis of variance model using IBM SPSS statistic 24. Fractional receptor densities correlating with half maximum initial binding rates were determined by non-linear regression analysis using GraphPad Prism 7.04 software. Significance analysis was based on two tailed unpaired t test or one way ANOVA followed by Tukey’s multiple comparisons test (GraphPad Prism 7.04).

## Supporting information

S1 Fig Geometric model of IAV virus–SA sensor interaction. (A) Streptavidin-coated (SA) biosensors contain 10^9^ biotin binding sites (Pall-ForteBio). SA tetramers carry four biotin binding sites, ordered two by two at opposing planes of the cubic structure [92]. Only two binding sites (spaced at 2.5 nm distance as determined by X-ray crystallography [93]) on one side of a surface-coated SA tetramer (25 nm center to center distance assuming regular hexagonal packaging) are assumed to have exposed biotin binding sites [93]. HA trimers are closely packaged on the virus surface (S3 Fig, in agreement with [94–97]) and the center to center distance has been determined at 12nm [94–97]. A fully loaded streptavidin can, in principle, form a bivalent interaction with an HA-trimer in which the SIA-binding sites are spaced at 4nm distance [94,97]. Lowering the receptor-density results in a non-homogenous sensor surface with streptavidins carrying 0, 1 or 2 receptor molecules. As a result, increasing amounts of surface-area will have a receptor density too low to bind virus at decreasing receptor concentrations thus contributing to the observed decrease in maximum binding levels and initial binding rate when lowering receptor density (Fig 1D and Fig 1E). (B) Labstrains PR8 and WSN_WT_ are spherical viruses with a diameter of ~ 100 nm (S3 Fig) [39–42]. When virus particles can be flattened for 0.2 times the radius 10% of the virus surface will be in contact with the sensor. (C) When 10% of the virus surface is in contact with the sensor, ~7 HA trimers can interact with receptor-loaded SA molecules at the virus-sensor contact interface (inner red circle). In principle two receptor molecules on a SA molecule can interact with an HA trimer but whether this occurs simultaneously will depend on the exact geometry of the specific glycan that was loaded. (D) At saturating levels of virus binding (hexagonal packaging) the majority of SA molecules are not present at the contact interface and therefore cannot be cleaved by NA without virus movement.
(TIF)

S2 Fig Overview of receptors and viruses used for BLI in this report. (A) Schematic representation of BLI sensors loaded with sialosides and virus particles. Biotinylated receptors (synthetic glycans or glycoproteins) were bound to SA sensors whereas Fc-tagged glycoproteins were bound to Protein A sensors. (B) Synthetic glycans used in this study. Purple diamond, yellow circle, blue rectangle and red triangle correspond with sialic acid (SIA), galactose (Gal), N-acetylglucosamine (GlcNAC) and fucose (Fuc), respectively. The linkage type between SIA and Gal is indicated. (C) Glycoprotein receptors used in this study. Expression of Fc-tagged (red) fetuin (yellow) in CHO k1 cells yields 3’N+O fetuin carrying exclusively α2,3-linked sialic acids on N-linked and O-linked glycans. Expression of fetuin in CHO 15B cells (deficient in N-acetylglucosamine transferase I) yields 3’O fetuin with sialylated O-linked glycans but immature N-linked glycans that are not sialylated. Wild type fetuin carries 3 N-linked glycans and 3 O-linked glycans. Expression of a fetuin-encoding plasmid in which the O-linked glycosylation sites are removed by site-directed mutagenesis yields 3’N fetuin upon expression in CHO k1 cells and asialo fetuin upon expression in CHO 15B cells. Biotinylated transferrin (6’N transferrin bt) is commercially available and carries two N-linked glycans with α2,6 SIAs [88,89]. Biotinylated fetuin was made by expressing a construct encoding a Bap-tag fused to fetuin that, by co-transfection with a plasmid carrying a biotinylation enzyme, yields C-terminally biotinylated 3’N+O fetuin (3’N+O fetuin bt) upon expression in CHO K1 cells. (D) Confirmation of SIA linkage-type specificity of glycoproteins using lectin binding. The glycoproteins were analyzed for linkage type specificity of their sialic acids using lectins MAL I (specific for SIAα2,3Galα1,4GlcNAc linkages abundantly present on N-linked glycans), MAL II (specific for SIAα2,3Galα1,3GalNAc linkages abundantly present on O-linked glycans), SNA (specific for SIAα2,6Galα1,4GlcNAc linkages abundantly present on N-linked glycans), and ECA (specific for terminal Galα1,4GlcNAc epitopes present on non-sialylated N-linked glycan antennae). (E) Viruses used for binding to receptor-loaded sensors are wild type PR8_MtSIN_, wild type WSN_WT_ and recombinant viruses carrying the HA encoding segments of PR8_MtSIN_ (WSN_HAMtSIN_) or PR8_CAM_ (PR8_CAM2,6_) in the background of seven WSN segments. PR8_CAM2,3_ is identical to PR8_CAM2,6_ except for a substitution (D190E) that was introduced in HA to obtain a shift from α2,6 to α2,3 linkage-type binding specificity. TX77_NAMtSIN_ carries the HA encoding segment of A/Bilthoven/1761/76 (H3N2) in the background of seven PR8_MtSIN_ segments [77].
(TIF)

S3 Fig Electron micrographs of influenza A virions stained by phosphotungstic acid (PTA). (A) Overview image of a field of PR8_MtSIN_ virions (Bar: 1.0μm)_._ (B-F) Representative images of single virus particles of PR8_MtSIN_, WSN_HAMtSIN_, WSN_WT_, PR8_CAM2,6_ and PR8_CAM2,3._ The large majority of all these virus particles are spherical particles with the diameters of about 100nm in agreement with the literature [39–42] (Bar: 200nm).
(TIF)

S4 Fig Association of PR8_MtSIN_ or PR8_CAM2,3_ for prolonged times or at different receptor densities. PR8_MtSIN_ (A) or PR8_CAM2,3_ (B) were bound for 240 min at the indicated concentrations to SA sensors maximally loaded with 3’SLN. (C) NA activity of PR8_MtSIN_ virus particles that were completely dissociated from a maximally loaded sensor (3’SLN receptor) by self-elution into 100 μl PBS was determined in comparison to 100 μl of 100 pM PR8_MtSIN_ used for initial loading using two-fold dilutions in a MUNANA assay. The results indicate that 0.71% of the virus particles present during the initial loading were associated to the sensor surface. This is close to the calculated maximal loading of 3.3E+07 spherical particles of 100nm diameter to the sensor, which corresponds to 0.55% of a 100 μl solution containing 100pM virus particles. (D, E) Biotinylated 3’SLN was loaded to the sensors at a density range as indicated in the figure (fractional loading of 1.0 corresponds to a sensor maximally loaded with receptor) followed by binding of 100 pM PR8_MtSIN_ (D) or PR8_CAM2,3_ (E). (F) Fractional virus association (virus association relative to the maximal virus binding level after 60 min) was plotted against fractional receptor loading. (G, H) PR8_CAM2,6_ was bound for 30 min at the indicated concentrations to SA sensors that were maximally loaded with 3’SLNLN (G) or 6’SLNLN (H). (I) The initial binding rates (*v*^*obs*^) for the curves obtained in (G) and (H) were calculated and the relative *v*^*obs*^ (3’SLNLN/6’SLNLN) at each virus concentration was determined and plotted.
(TIF)

S5 Fig Quantification of sensor-regeneration efficiency. (A) Biotinylated synthetic glycans 3’ or 6’ SLNLN, or biotinylated 3’N+O fetuin bt and 6’N transferrin bt (S2 Fig) were loaded to streptavidin sensors, after which sensor-regeneration were tested followed by binding of corresponding lectins and virus from the same well. Fractional binding of 1.0 corresponds to the average binding levels of each lectins and viruses. (B) Binding of PR8_MtSIN_ to sensors loaded with biotinylated 3’N+O fetuin bt. (C) After virus binding shown in S5B Fig, sensors were regenerated and PR8_MtSIN_-specific antibody (03/242 from NIBSC) was used for detection of virus binding to the regenerated sensor surface. No antibody binding was detected after regeneration, indicated efficient removal of virus from the sensor by regeneration.
(TIF)

S6 Fig Quantification of binding specificity to N-linked or O-linked glycoproteins. (A-D) Fc-tagged fetuin specifically engineered and expressed to carry either exclusively N-linked or O-linked glycans, a mixture of both glycan types (N+O), or no sialylated glycans at all (asialo fetuin) (S2 Fig) was loaded to Protein A-coated sensors to maximum levels after which binding of 100 pM PR8_MtSIN_ and PR8_CAM2,3_ was performed. 10 μM OC was present during virion binding. The initial binding rates were calculated and plotted in the bar diagram inserts. Whereas both PR8_MtSIN_ and PR8_CAM2,3_ bound to fetuin containing N-glycans (A and B), only PR8_CAM2,3_ was able to bind fetuin containing O-glycans, albeit at a 6-fold lower initial binding rate than to N-glycosylated fetuin (C). When N- and O-linked glycans are both present (A), the initial binding rate seems to be determined by the stronger binding to N-linked glycans as binding of PR8_CAM2,3_ is not accelerated despite its ability to bind α2,3 sialylated O-linked glycans. (E-G) Confirmation of presence of N- and/or O-glycan on recombinant fetuin. The glycoproteins were treated with PNGase F (NEB) for 4 hours at 37°C under non-denaturing conditions, which effectively removes almost all N-linked oligosaccharides from glycoproteins. The glycoproteins were analyzed for linkage type specificity using lectins MAL I (specific for SIAα2,3Galα1,4GlcNAc linkages abundantly present on N-linked glycans), MAL II (specific for SIAα2,3Galα1,3GalNAc linkages abundantly present on O-linked glycans), and ECA (specific for terminal Galα1,4GlcNAc epitopes present on non-sialylated N-linked glycan antennae). (E) After PNGase F treatment, the binding level of MAL I to 3’ N+O (blue) and 3’ N fetuins (red) significantly reduced, whereas the binding level to 3’ O fetuin (green) remains the same, indicating the presence of sialylated N-linked oligosaccharides specifically on 3’ N+O and 3’ N fetuin. (F) After PNGase F treatment, the binding level of ECA to 3’ N+O (blue) and 3’ N fetuins (red) dramatically decreased, whereas the binding level to 3’ O fetuin (green) remains the same, indicating the presence of non-sialylated N-linked oligosaccharides specifically on 3’ N+O and 3’ N fetuin. (G) Similar binding of MAL II to glycoproteins was observed after treatment with PNGase F, indicating that O-linked oligosaccharides were not removed by PNGase F treatment.
(TIF)

S7 Fig Quantification of virus particle number and HA and NA content. Reliable determination of *v*^*obs*^ depends on precise determination of virus concentration. Hemagglutination titers or infectious titers do not reveal absolute or relative particle of different viruses. Quantitative PCR or Western blotting (usually targeting NP) are sensitive to variation caused by RNA or protein contamination of virus preparations and, when comparing different viruses, to differences in specificity of probes or antibodies. Therefore, densitometric quantification of the polymerase content of a virus preparation was used, assuming that every particle carries eight genome segments, each attached to a heterotrimeric complex of the three polymerase subunits PB1, PB2 and PA. (A) Silver-stained SDS-PAGE gel showing the separation of a MW marker and a PR8_MtSIN_ virus stock. (B) Silver-stained region showing the polymerase complex (PB1, PB2 and PA appearing as a single band due to similar size) of a dilution series of five virus stocks. Densitometric quantification was calibrated using a dilution series of the molecular marker shown in (A). (C) Calculated concentration of virus stocks (pM) assuming a MW of 250 kDa for the PB1/PB2/PA complex. (D) Western blot of the HA0 band of a concentration series of five viruses using monoclonal antibody FI6 recognizing a universally conserved epitope localized in the stem of HA. (E) Densitometric quantification of HA trimers (nM) was calibrated using by Western blotting of a concentration series of PR8_MtSIN_ and WSN_WT_ HA proteins expressed in HEK293S-GNTI(-) cells [33]. (F) Number of HA trimers per virus particle as derived from (C) and (E). The obtained numbers/particle fit well to numbers obtained by different methods by others [98,99]. (G, H, I) Quantification of NA by similar procedures as applied for HA in panel D-F. Antibodies GT288-GTX629696 (WSN) and N1-7D3 (PR8) were used and quantification was calibrated using a Western blot of a standard concentration series of recombinant soluble NA of PR8_MtSIN_ and WSN expressed in HEK293T cells run in parallel. The NA proteins were expressed similarly are described previously [100]. (J) NA activity of PR8_MtSIN_ and WSN_WT_ virus particles and expressed recombinant NA soluble tetramers was determined using a two-fold dilution series in a soluble substrate NA activity assay (MUNANA assay). The activity of recombinant WSN NA was 5.4-fold lower than of PR8_*MtSIN*_ NA, whereas the NA activity of the cognate virus particles showed a 31.4-fold lower activity for WSN_WT_ particles. This difference is explained by the 6.4-fold higher incorporation level of NA in PR8_MtSIN_ particles as determined by Western blotting (G-I). The HA content of both viruses was similar (D-F). (K) HA/NA ratio was determined from panels F and I. (C-K) Error bars indicate SD.
(TIF)

S8 Fig **Comparison of the elution rates from 3’N fetuin and 6’N transferrin bt for WSN**_**WT**_ **(A) and PR8**_**CAM2,6**_ **(B) carrying the same NA (from WSN) but a different HA.** Both viruses bind at similar, but relatively low, rate to 6’N transferrin bt (Fig 2E) whereas WSN_WT_ displays a ~3-fold faster binding rate to 3’N fetuin than PR8_CAM2,6_ (Fig 2D). HA clearly affects the self-elution rate as, in combination with the same NA, self-elution from 3’N fetuin is much more efficient in companion of the weaker binding HA of PR8_CAM2,6_ (B) than in companion of the stronger binding HA of WSN_WT_ (A). Self-elution rates from 6’N transferrin bt are more similar for both viruses. Thus, whereas α2,3 versus α2,6 SIA specificity of NA is seemingly opposite for PR8_CAM2,6_ and WSN_WT_, this is not caused by the NA itself (which is identical for both viruses) but by differences in their HAs.
(TIF)

S9 Fig The NA activity-driven self-elution rate depends on virus binding level. Four viruses (A-B, WSN_HAMtSIN_; C-D, WSN_WT_; E-F, PR8_CAM2,6_; G-H, PR8_CAM2,3_) all carrying the same NA (NA_WSN_) but a different HA were bound to Fc-tagged 3’N fetuin loaded to maximum level at three virus concentrations as indicated in the panels. Viruses were bound for 15 min in presence (A, C, E, G) or absence (B, D, F, H) of 10 μM OC. In absence of OC, ongoing receptor cleavage reduces receptor density in time and thus the binding rate of additional virus particles, resulting in bending of the curves. As receptor cleavage by NA is in competition with receptor binding by HA, the weakest binder (PR8_CAM2,6_), which is assisted most in initial binding rate by NA (Fig 5G), will also suffer most from receptor destruction by its NA and as a consequence display a binding curve that bends down fastest in absence of OC (F).
(TIF)
