# Supplementary figures and images for: Kinetic analysis of the influenza A virus HA/NA balance reveals contribution of NA to virus-receptor binding and NA-dependent rolling on receptor-containing surfaces
Source: PLoS Pathog. 2018 Aug 13;14(8):e1007233. doi: 10.1371/journal.ppat.1007233 (PMC6107293; doi:10.1371/journal.ppat.1007233)

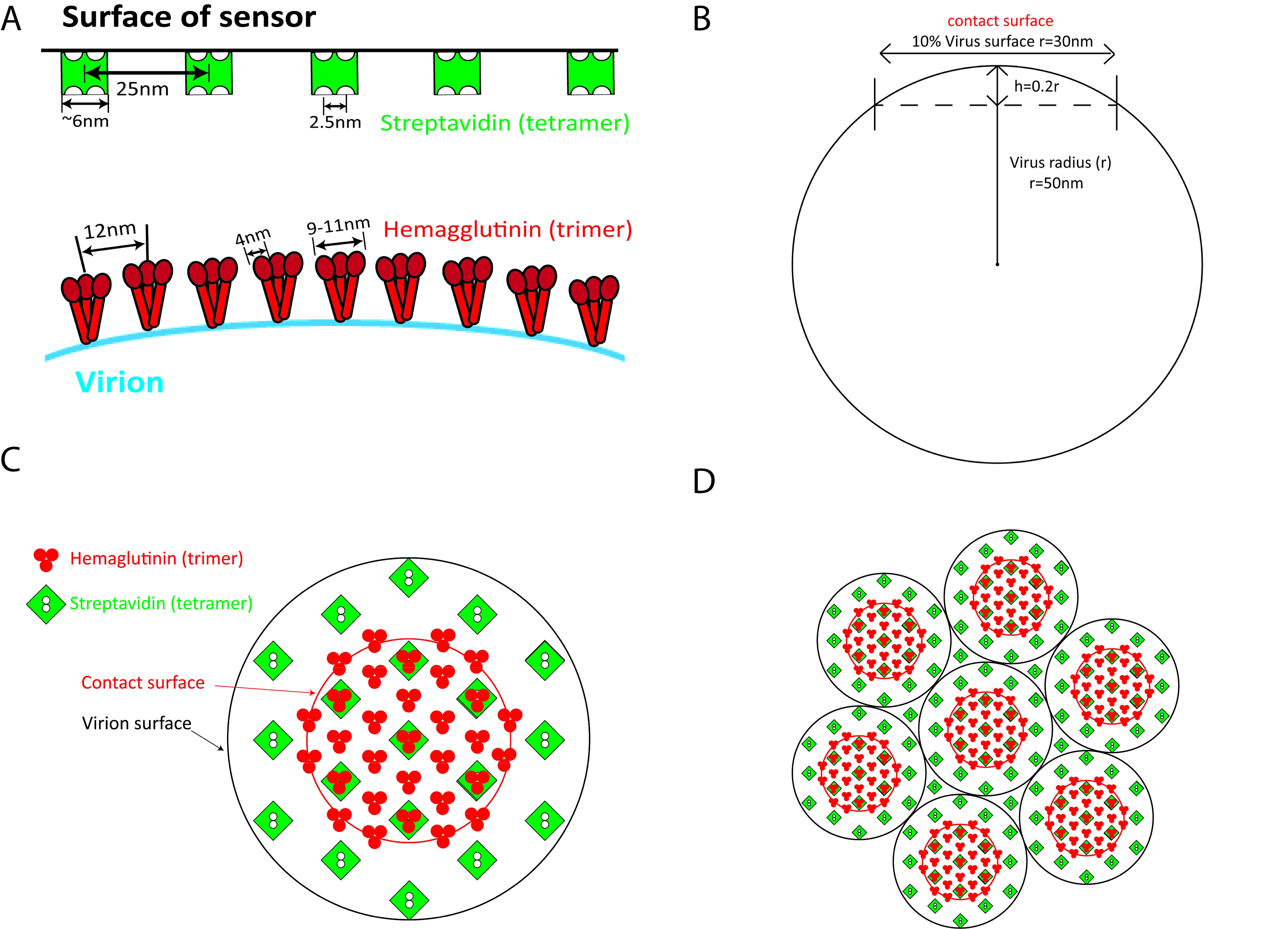

Supplement: S1 Fig — (A) Streptavidin-coated (SA) biosensors contain 109 biotin binding sites (Pall-ForteBio). SA tetramers carry four biotin binding sites, ordered two by two at opposing planes of the cubic structure [92]. Only two binding sites (spaced at 2.5 nm distance as determined by X-ray crystallography [93]) on one side of a surface-coated SA tetramer (25 nm center to center distance assuming regular hexagonal packaging) are assumed to have exposed biotin binding sites [93]. HA trimers are closely packaged on the virus surface (S3 Fig, in agreement with [94–97]) and the center to center distance has been determined at 12nm [94–97]. A fully loaded streptavidin can, in principle, form a bivalent interaction with an HA-trimer in which the SIA-binding sites are spaced at 4nm distance [94,97]. Lowering the receptor-density results in a non-homogenous sensor surface with streptavidins carrying 0, 1 or 2 receptor molecules. As a result, increasing amounts of surface-area will have a receptor density too low to bind virus at decreasing receptor concentrations thus contributing to the observed decrease in maximum binding levels and initial binding rate when lowering receptor density (Fig 1D and Fig 1E). (B) Labstrains PR8 and WSNWT are spherical viruses with a diameter of ~ 100 nm (S3 Fig) [39–42]. When virus particles can be flattened for 0.2 times the radius 10% of the virus surface will be in contact with the sensor. (C) When 10% of the virus surface is in contact with the sensor, ~7 HA trimers can interact with receptor-loaded SA molecules at the virus-sensor contact interface (inner red circle). In principle two receptor molecules on a SA molecule can interact with an HA trimer but whether this occurs simultaneously will depend on the exact geometry of the specific glycan that was loaded. (D) At saturating levels of virus binding (hexagonal packaging) the majority of SA molecules are not present at the contact interface and therefore cannot be cleaved by NA without virus movement. [file ppat.1007233.s001.tif]

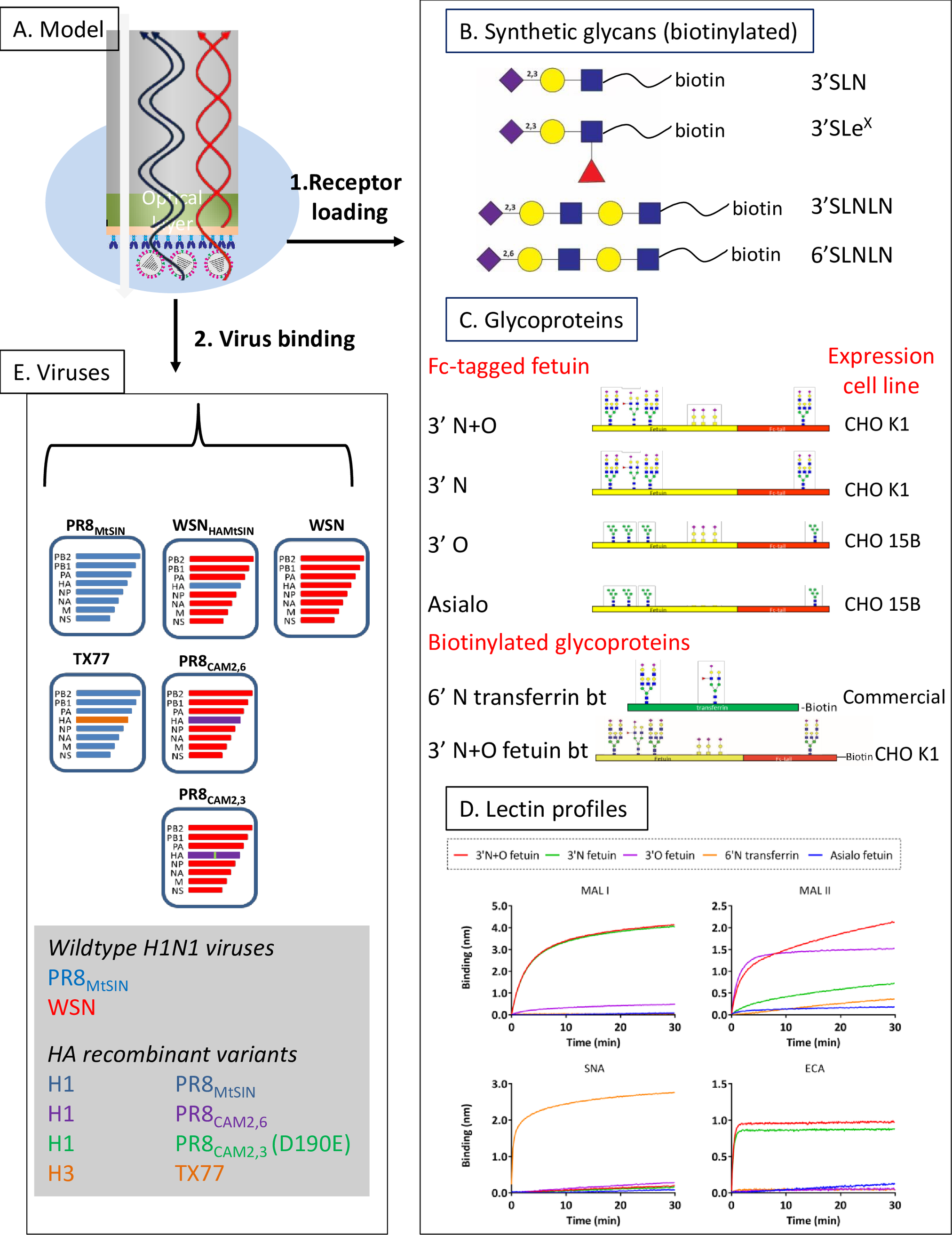

Supplement: S2 Fig — (A) Schematic representation of BLI sensors loaded with sialosides and virus particles. Biotinylated receptors (synthetic glycans or glycoproteins) were bound to SA sensors whereas Fc-tagged glycoproteins were bound to Protein A sensors. (B) Synthetic glycans used in this study. Purple diamond, yellow circle, blue rectangle and red triangle correspond with sialic acid (SIA), galactose (Gal), N-acetylglucosamine (GlcNAC) and fucose (Fuc), respectively. The linkage type between SIA and Gal is indicated. (C) Glycoprotein receptors used in this study. Expression of Fc-tagged (red) fetuin (yellow) in CHO k1 cells yields 3’N+O fetuin carrying exclusively α2,3-linked sialic acids on N-linked and O-linked glycans. Expression of fetuin in CHO 15B cells (deficient in N-acetylglucosamine transferase I) yields 3’O fetuin with sialylated O-linked glycans but immature N-linked glycans that are not sialylated. Wild type fetuin carries 3 N-linked glycans and 3 O-linked glycans. Expression of a fetuin-encoding plasmid in which the O-linked glycosylation sites are removed by site-directed mutagenesis yields 3’N fetuin upon expression in CHO k1 cells and asialo fetuin upon expression in CHO 15B cells. Biotinylated transferrin (6’N transferrin bt) is commercially available and carries two N-linked glycans with α2,6 SIAs [88,89]. Biotinylated fetuin was made by expressing a construct encoding a Bap-tag fused to fetuin that, by co-transfection with a plasmid carrying a biotinylation enzyme, yields C-terminally biotinylated 3’N+O fetuin (3’N+O fetuin bt) upon expression in CHO K1 cells. (D) Confirmation of SIA linkage-type specificity of glycoproteins using lectin binding. The glycoproteins were analyzed for linkage type specificity of their sialic acids using lectins MAL I (specific for SIAα2,3Galα1,4GlcNAc linkages abundantly present on N-linked glycans), MAL II (specific for SIAα2,3Galα1,3GalNAc linkages abundantly present on O-linked glycans), SNA (specific for SIAα2,6Galα1,4GlcNAc li [file ppat.1007233.s002.tif]

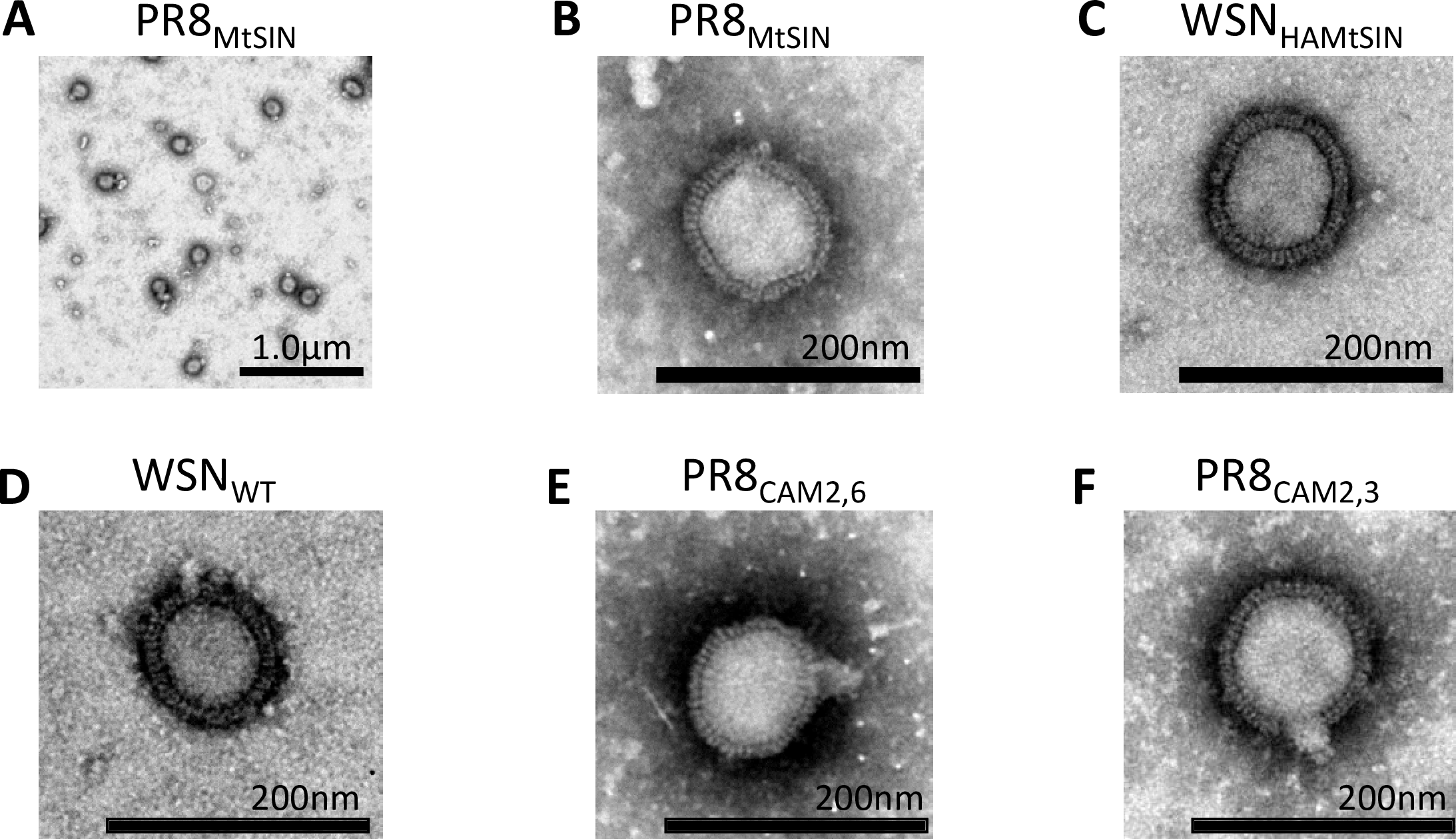

Supplement: S3 Fig — (A) Overview image of a field of PR8MtSIN virions (Bar: 1.0μm). (B-F) Representative images of single virus particles of PR8MtSIN, WSNHAMtSIN, WSNWT, PR8CAM2,6 and PR8CAM2,3. The large majority of all these virus particles are spherical particles with the diameters of about 100nm in agreement with the literature [39–42] (Bar: 200nm). (TIF) [file ppat.1007233.s003.tif]

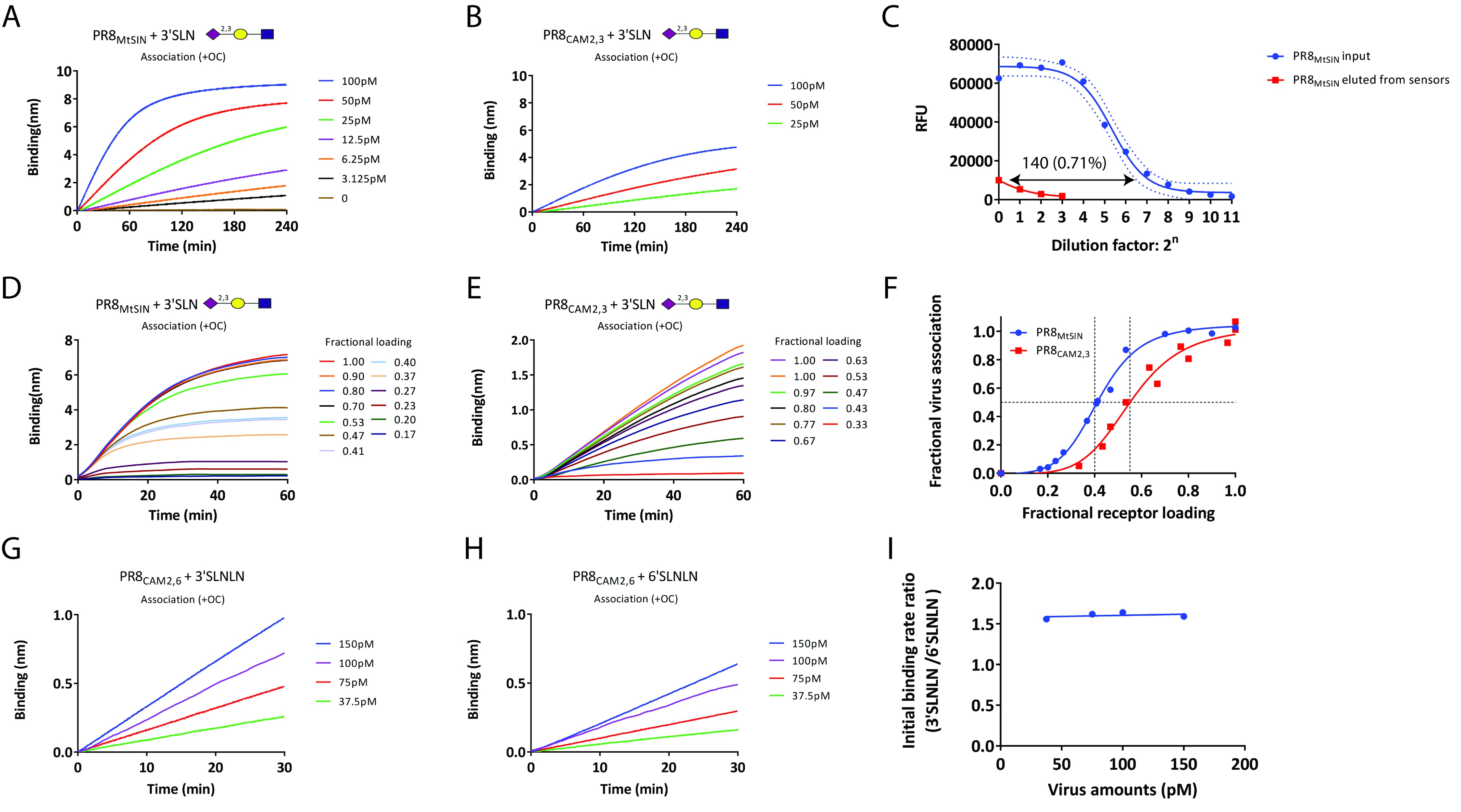

Supplement: S4 Fig — PR8MtSIN (A) or PR8CAM2,3 (B) were bound for 240 min at the indicated concentrations to SA sensors maximally loaded with 3’SLN. (C) NA activity of PR8MtSIN virus particles that were completely dissociated from a maximally loaded sensor (3’SLN receptor) by self-elution into 100 μl PBS was determined in comparison to 100 μl of 100 pM PR8MtSIN used for initial loading using two-fold dilutions in a MUNANA assay. The results indicate that 0.71% of the virus particles present during the initial loading were associated to the sensor surface. This is close to the calculated maximal loading of 3.3E+07 spherical particles of 100nm diameter to the sensor, which corresponds to 0.55% of a 100 μl solution containing 100pM virus particles. (D, E) Biotinylated 3’SLN was loaded to the sensors at a density range as indicated in the figure (fractional loading of 1.0 corresponds to a sensor maximally loaded with receptor) followed by binding of 100 pM PR8MtSIN (D) or PR8CAM2,3 (E). (F) Fractional virus association (virus association relative to the maximal virus binding level after 60 min) was plotted against fractional receptor loading. (G, H) PR8CAM2,6 was bound for 30 min at the indicated concentrations to SA sensors that were maximally loaded with 3’SLNLN (G) or 6’SLNLN (H). (I) The initial binding rates (vobs) for the curves obtained in (G) and (H) were calculated and the relative vobs (3’SLNLN/6’SLNLN) at each virus concentration was determined and plotted. (TIF) [file ppat.1007233.s004.tif]

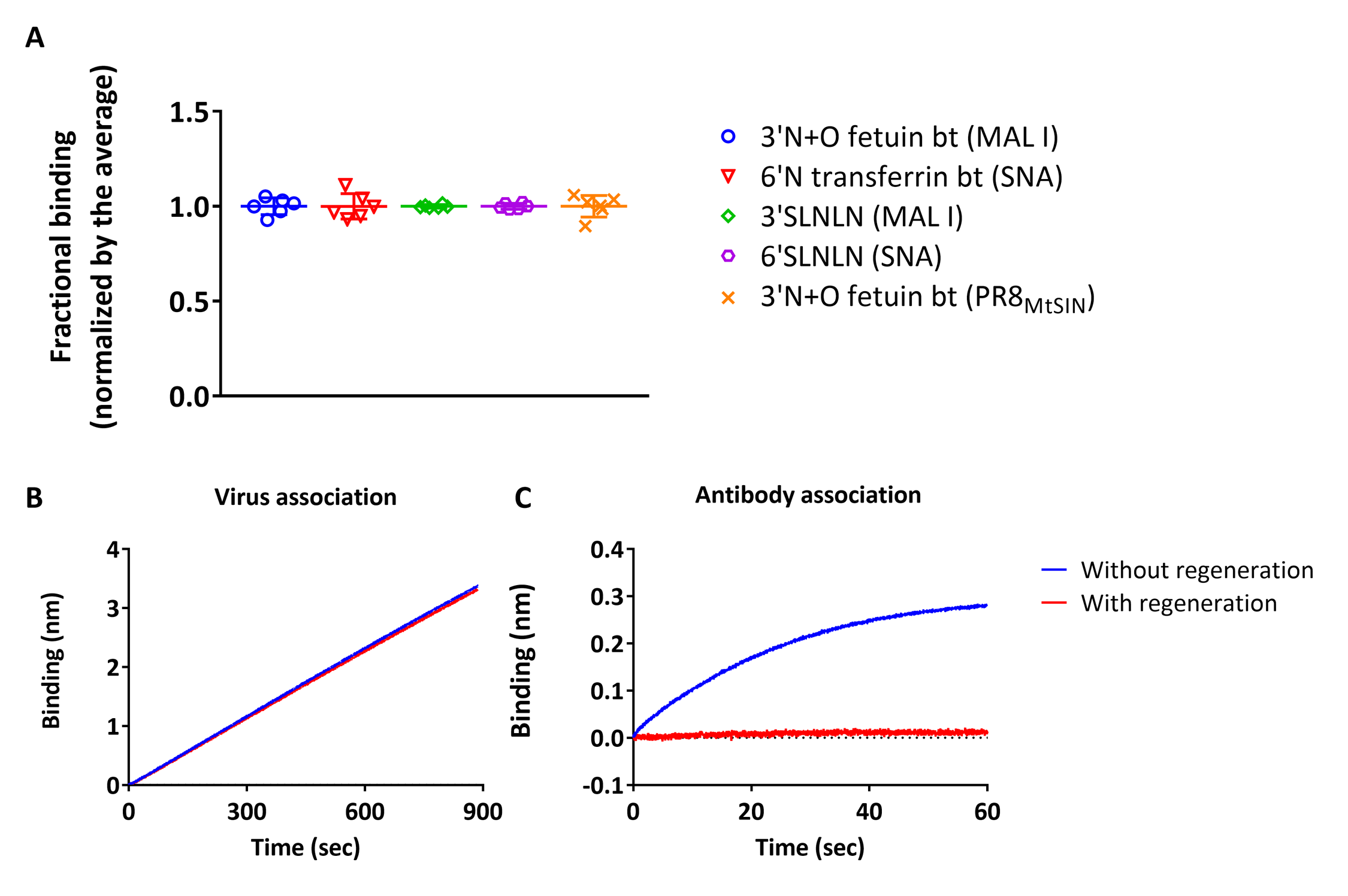

Supplement: S5 Fig — (A) Biotinylated synthetic glycans 3’ or 6’ SLNLN, or biotinylated 3’N+O fetuin bt and 6’N transferrin bt (S2 Fig) were loaded to streptavidin sensors, after which sensor-regeneration were tested followed by binding of corresponding lectins and virus from the same well. Fractional binding of 1.0 corresponds to the average binding levels of each lectins and viruses. (B) Binding of PR8MtSIN to sensors loaded with biotinylated 3’N+O fetuin bt. (C) After virus binding shown in S5B Fig, sensors were regenerated and PR8MtSIN-specific antibody (03/242 from NIBSC) was used for detection of virus binding to the regenerated sensor surface. No antibody binding was detected after regeneration, indicated efficient removal of virus from the sensor by regeneration. (TIF) [file ppat.1007233.s005.tif]

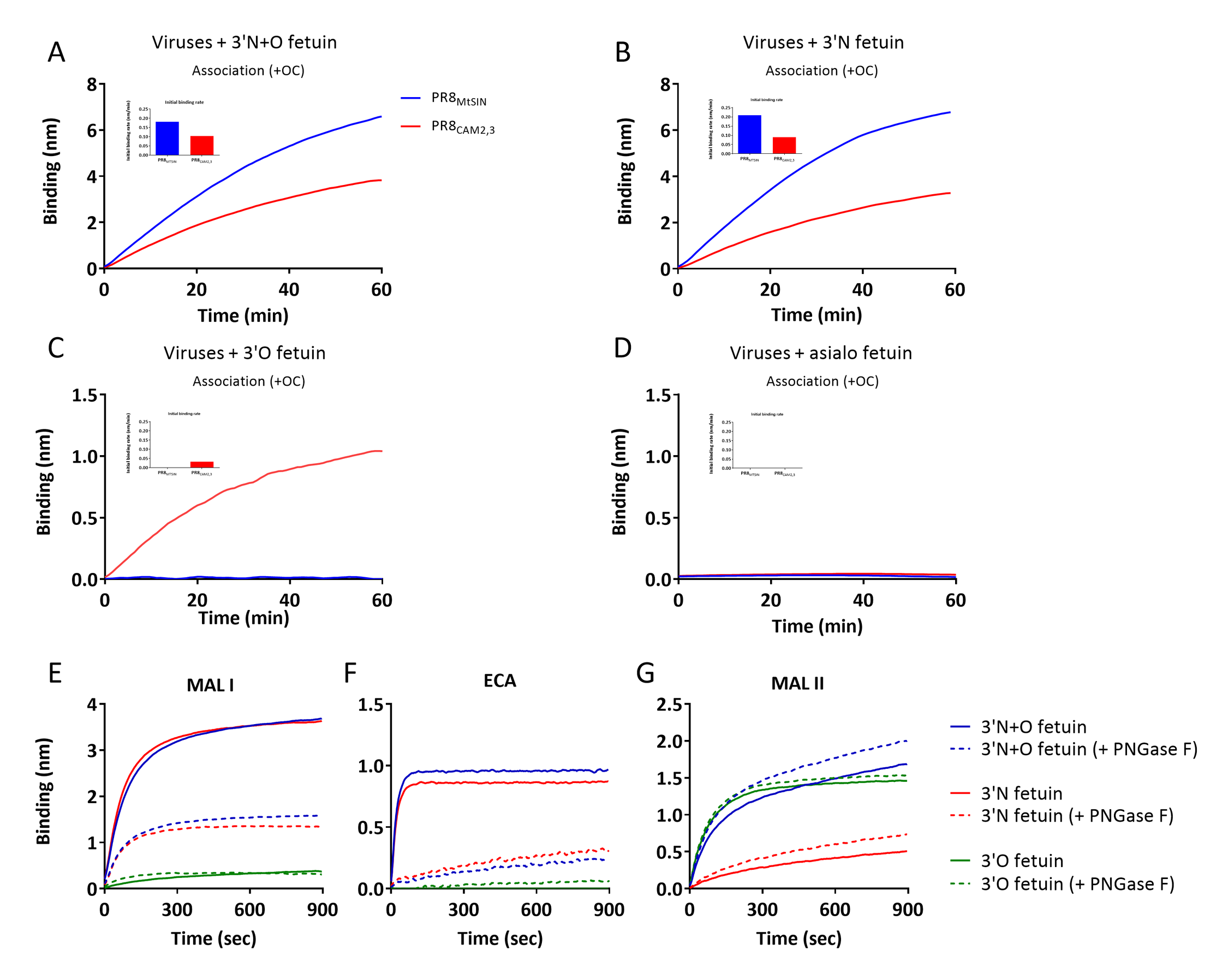

Supplement: S6 Fig — (A-D) Fc-tagged fetuin specifically engineered and expressed to carry either exclusively N-linked or O-linked glycans, a mixture of both glycan types (N+O), or no sialylated glycans at all (asialo fetuin) (S2 Fig) was loaded to Protein A-coated sensors to maximum levels after which binding of 100 pM PR8MtSIN and PR8CAM2,3 was performed. 10 μM OC was present during virion binding. The initial binding rates were calculated and plotted in the bar diagram inserts. Whereas both PR8MtSIN and PR8CAM2,3 bound to fetuin containing N-glycans (A and B), only PR8CAM2,3 was able to bind fetuin containing O-glycans, albeit at a 6-fold lower initial binding rate than to N-glycosylated fetuin (C). When N- and O-linked glycans are both present (A), the initial binding rate seems to be determined by the stronger binding to N-linked glycans as binding of PR8CAM2,3 is not accelerated despite its ability to bind α2,3 sialylated O-linked glycans. (E-G) Confirmation of presence of N- and/or O-glycan on recombinant fetuin. The glycoproteins were treated with PNGase F (NEB) for 4 hours at 37°C under non-denaturing conditions, which effectively removes almost all N-linked oligosaccharides from glycoproteins. The glycoproteins were analyzed for linkage type specificity using lectins MAL I (specific for SIAα2,3Galα1,4GlcNAc linkages abundantly present on N-linked glycans), MAL II (specific for SIAα2,3Galα1,3GalNAc linkages abundantly present on O-linked glycans), and ECA (specific for terminal Galα1,4GlcNAc epitopes present on non-sialylated N-linked glycan antennae). (E) After PNGase F treatment, the binding level of MAL I to 3’ N+O (blue) and 3’ N fetuins (red) significantly reduced, whereas the binding level to 3’ O fetuin (green) remains the same, indicating the presence of sialylated N-linked oligosaccharides specifically on 3’ N+O and 3’ N fetuin. (F) After PNGase F treatment, the binding level of ECA to 3’ N+O (blue) and 3’ N fetuins (red) dramatically decreased, whereas the binding lev [file ppat.1007233.s006.tif]

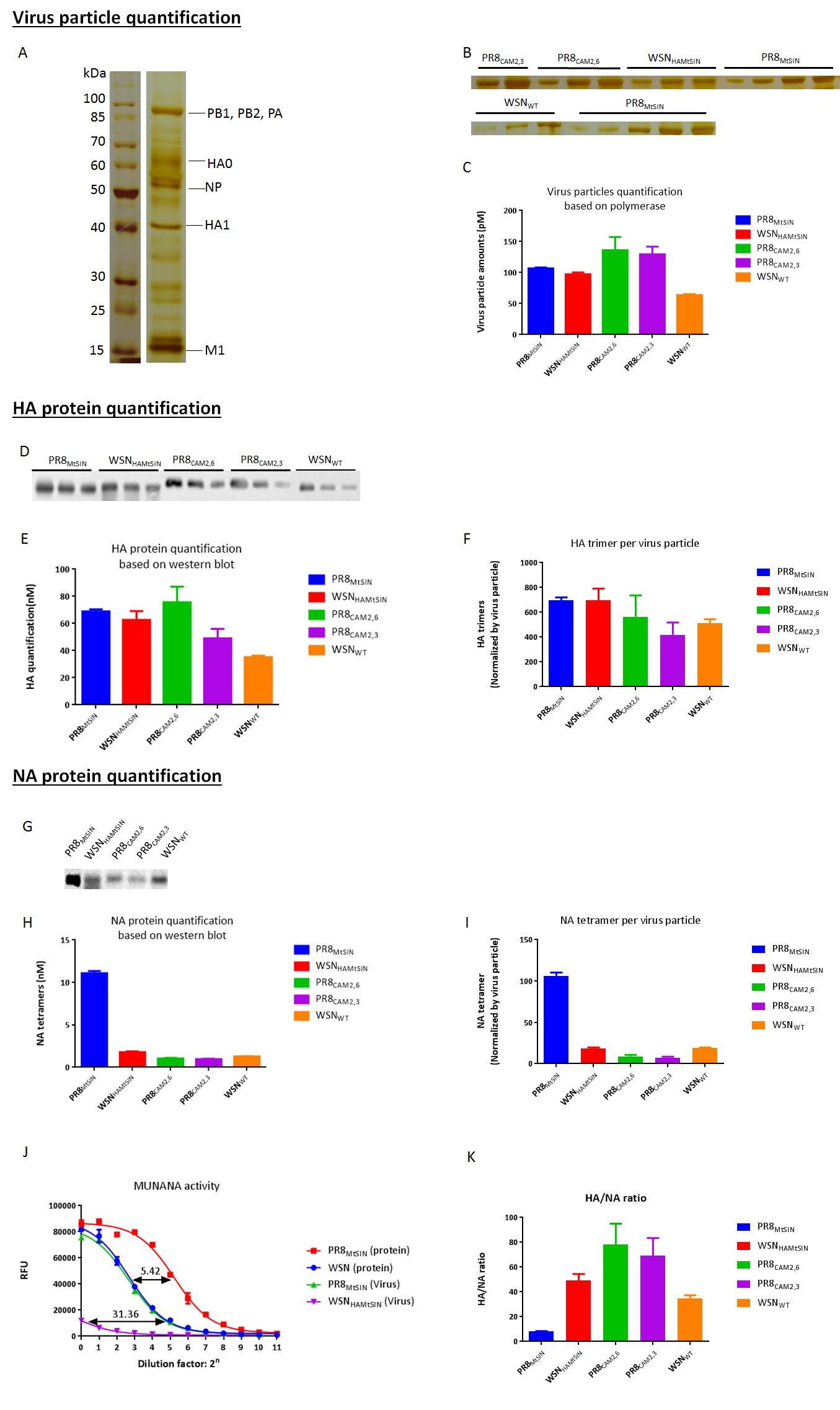

Supplement: S7 Fig — Reliable determination of vobs depends on precise determination of virus concentration. Hemagglutination titers or infectious titers do not reveal absolute or relative particle of different viruses. Quantitative PCR or Western blotting (usually targeting NP) are sensitive to variation caused by RNA or protein contamination of virus preparations and, when comparing different viruses, to differences in specificity of probes or antibodies. Therefore, densitometric quantification of the polymerase content of a virus preparation was used, assuming that every particle carries eight genome segments, each attached to a heterotrimeric complex of the three polymerase subunits PB1, PB2 and PA. (A) Silver-stained SDS-PAGE gel showing the separation of a MW marker and a PR8MtSIN virus stock. (B) Silver-stained region showing the polymerase complex (PB1, PB2 and PA appearing as a single band due to similar size) of a dilution series of five virus stocks. Densitometric quantification was calibrated using a dilution series of the molecular marker shown in (A). (C) Calculated concentration of virus stocks (pM) assuming a MW of 250 kDa for the PB1/PB2/PA complex. (D) Western blot of the HA0 band of a concentration series of five viruses using monoclonal antibody FI6 recognizing a universally conserved epitope localized in the stem of HA. (E) Densitometric quantification of HA trimers (nM) was calibrated using by Western blotting of a concentration series of PR8MtSIN and WSNWT HA proteins expressed in HEK293S-GNTI(-) cells [33]. (F) Number of HA trimers per virus particle as derived from (C) and (E). The obtained numbers/particle fit well to numbers obtained by different methods by others [98,99]. (G, H, I) Quantification of NA by similar procedures as applied for HA in panel D-F. Antibodies GT288-GTX629696 (WSN) and N1-7D3 (PR8) were used and quantification was calibrated using a Western blot of a standard concentration series of recombinant soluble NA of PR8MtSIN and WSN expressed i [file ppat.1007233.s007.tif]

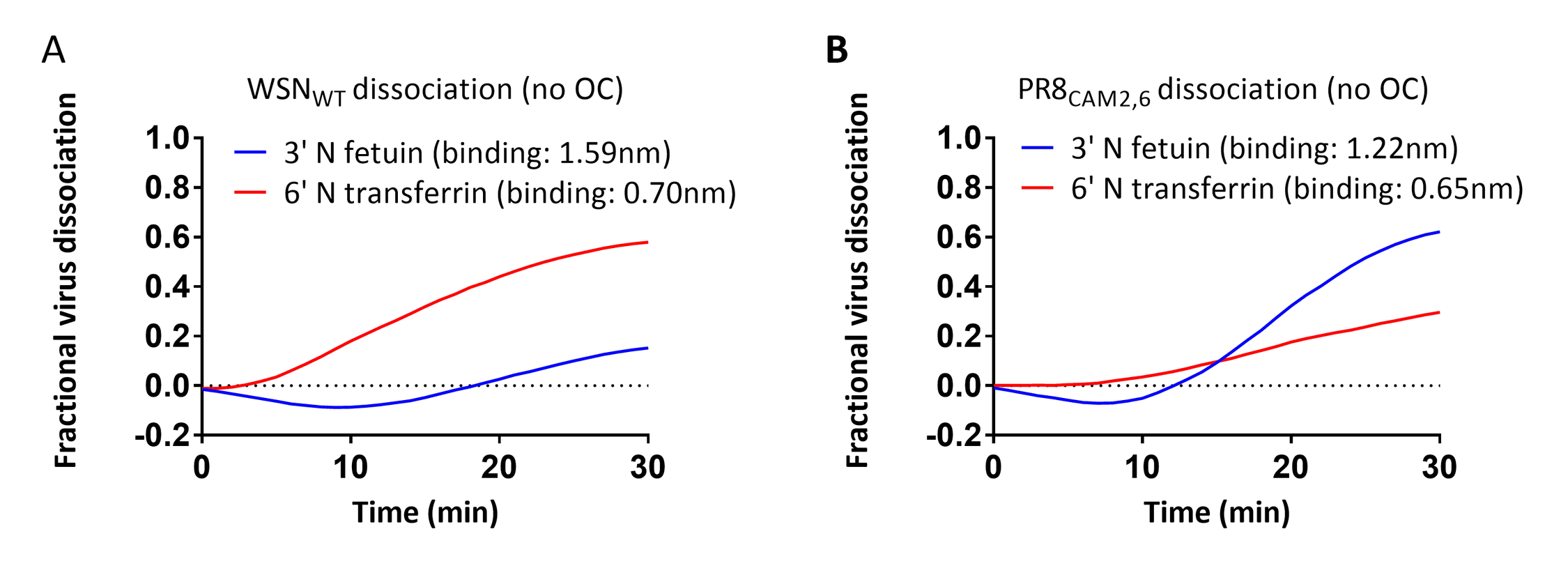

Supplement: S8 Fig — Comparison of the elution rates from 3’N fetuin and 6’N transferrin bt for WSNWT (A) and PR8CAM2,6 (B) carrying the same NA (from WSN) but a different HA. Both viruses bind at similar, but relatively low, rate to 6’N transferrin bt (Fig 2E) whereas WSNWT displays a ~3-fold faster binding rate to 3’N fetuin than PR8CAM2,6 (Fig 2D). HA clearly affects the self-elution rate as, in combination with the same NA, self-elution from 3’N fetuin is much more efficient in companion of the weaker binding HA of PR8CAM2,6 (B) than in companion of the stronger binding HA of WSNWT (A). Self-elution rates from 6’N transferrin bt are more similar for both viruses. Thus, whereas α2,3 versus α2,6 SIA specificity of NA is seemingly opposite for PR8CAM2,6 and WSNWT, this is not caused by the NA itself (which is identical for both viruses) but by differences in their HAs. (TIF) [file ppat.1007233.s008.tif]

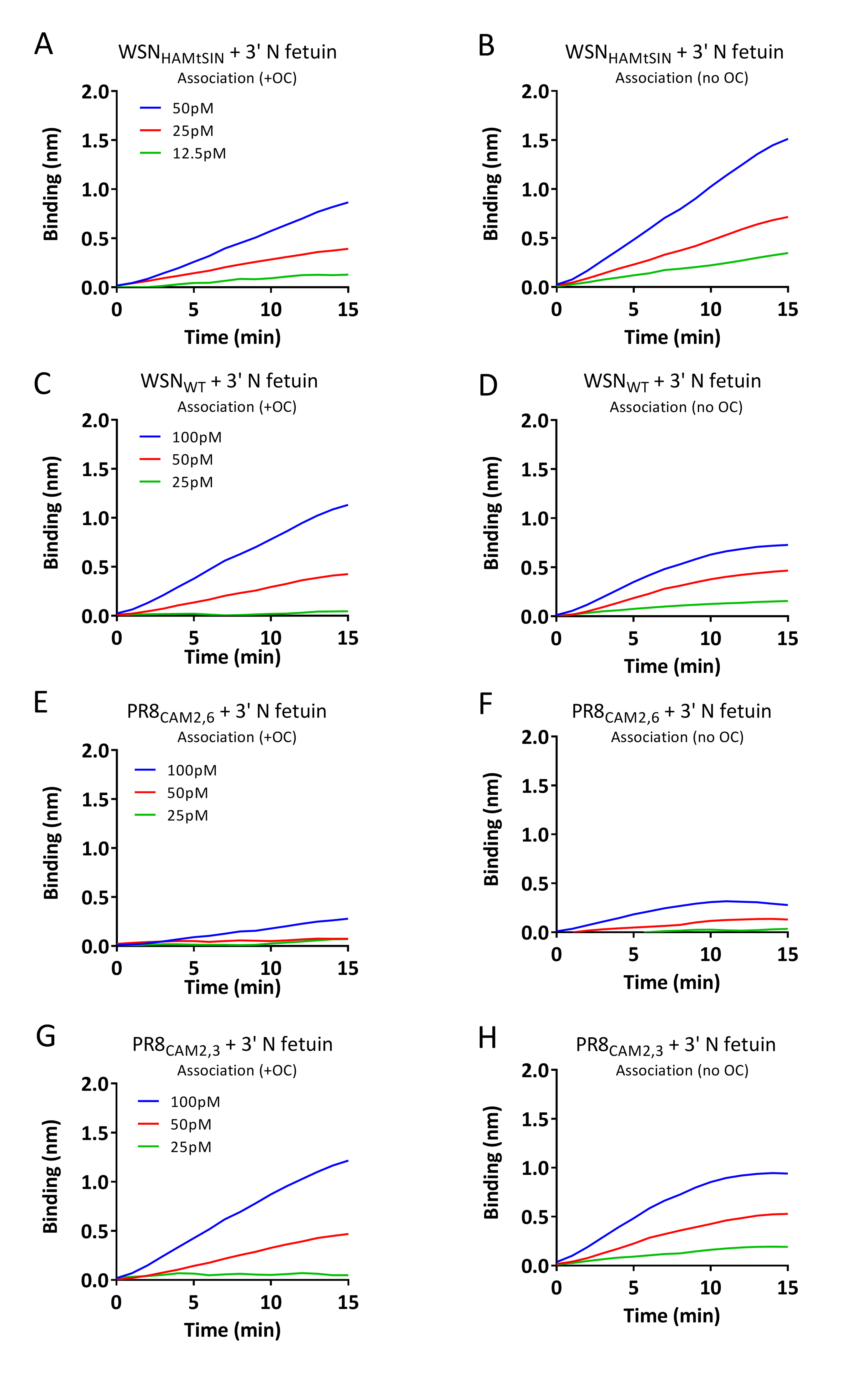

Supplement: S9 Fig — Four viruses (A-B, WSNHAMtSIN; C-D, WSNWT; E-F, PR8CAM2,6; G-H, PR8CAM2,3) all carrying the same NA (NAWSN) but a different HA were bound to Fc-tagged 3’N fetuin loaded to maximum level at three virus concentrations as indicated in the panels. Viruses were bound for 15 min in presence (A, C, E, G) or absence (B, D, F, H) of 10 μM OC. In absence of OC, ongoing receptor cleavage reduces receptor density in time and thus the binding rate of additional virus particles, resulting in bending of the curves. As receptor cleavage by NA is in competition with receptor binding by HA, the weakest binder (PR8CAM2,6), which is assisted most in initial binding rate by NA (Fig 5G), will also suffer most from receptor destruction by its NA and as a consequence display a binding curve that bends down fastest in absence of OC (F). (TIF) [file ppat.1007233.s009.tif]
